# Exchange of CO_2_ with CO as Reactant Switches Selectivity in Photoreduction on Co−ZrO_2_ from C_1–3_ Paraffin to Small Olefins

**DOI:** 10.1002/anie.202412090

**Published:** 2024-11-02

**Authors:** Tarik Loumissi, Rento Ishii, Keisuke Hara, Tomoki Oyumi, Ikki Abe, Chongxu Li, Hongwei Zhang, Rumiko Hirayama, Kaori Niki, Takaomi Itoi, Yasuo Izumi

**Affiliations:** ^1^ Department of Chemistry Graduate School of Science Chiba University Yayoi 1–33, Inage-ku Chiba 263-8522 Japan; ^2^ Department of Mechanical Engineering Graduate School of Engineering Chiba University Yayoi 1–33, Inage-ku Chiba 263-8522 Japan

**Keywords:** Photocatalyst, Cobalt, Carbon dioxide, Paraffin, Ethene

## Abstract

Photocatalytic reduction of CO_2_ into C_2,3_ hydrocarbons completes a C‐neutral cycle. The reaction pathways of photocatalytic generation of C_2,3_ paraffin and C_2_H_4_ from CO_2_ are mostly unclear. Herein, a Co^0^−ZrO_2_ photocatalyst converted CO_2_ into C_1–3_ paraffin, while selectively converting CO into C_2_H_4_ and C_3_H_6_ (6.0±0.6 μmol h^−1^ g_cat_^−1^, 70 mol %) only under UV/Visible light. The photocatalytic cycle was conducted under ^13^CO and H_2_, with subsequent evacuation and flushing with CO. This iterative process led to an increase in the population of C_2_H_4_ and C_3_H_6_ up to 61–87 mol %, attributed to the accumulation of CH_2_ species at the interface between Co^0^ nanoparticles and the ZrO_2_ surface. CO_2_ adsorbed onto the O vacancies of the ZrO_2_ surface, with resulting COH species undergoing hydrogenation on the Co^0^ surface to yield C_1–3_ paraffin using either H_2_ or H_2_O (g, l) as the reductant. In contrast, CO adsorbed on the Co^0^ surface, converted to HCOH species, and then split into CH and OH species at the Co and O vacancy sites on ZrO_2_, respectively. This comprehensive study elucidates intricate photocatalytic pathways governing the transformation of CO_2_ into paraffin and CO to olefins.

## Graphical abstract

Pathways from CO_2_ to paraffin while from CO to C_2_H_4_ and C_3_H_6_ using Co−ZrO_2_ photocatalyst were switched. CO_2_ at the O vacancy on ZrO_2_ transformed into COH and hopped onto Co^0^ to be paraffin while CO and H competed on Co^0^ leading to preferable coupling of CH_2_ to olefins.

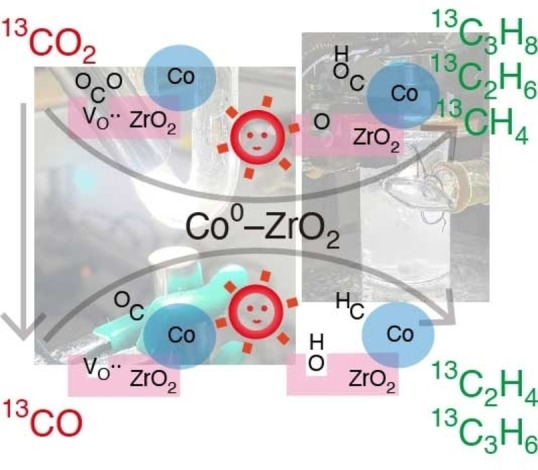

## Introduction

In contrast to the irreversible consumption of fossil fuels and raw materials, the conversion of CO_2_ reduction into fuels and/or valuable chemicals using a sustainable energy represents a pivotal step toward establishing a new carbon‐neutral cycle.[^1^, ^2^] Photocatalytic CO_2_ reduction offers a direct and simple approach; however, the range of products has been limited to CO, CH_4_, and CH_3_OH,[^1^, ^2^, ^3^] unlike the electrochemical production of a broad spectrum including formate,^[4]^ C_2_H_6_, C_2_H_4_,[^4^, ^5^] CH_3_CHO,^[4]^ C_2_H_5_OH,[^4^, ^5^, ^6^] C_3_H_8_, C_3_H_6_, C_3_H_7_OH,[^4^, ^6^] acetate,[^5^, ^6^] and oxalate^[5]^ from CO_2_ and/or CO, facilitated by concentrated electrons supplied from electricity. The economically viable nature that formed C_2_ and C_3_ hydrocarbons (HCs) derived from photocatalytic CO_2_ reduction (Table S1) has emerged as key chemicals (0.9–8 $ kg^−1^) compared to CO and CH_4_ (0.06–0.18 $ kg^−1^).^[3]^ Various catalysts have been explored for this purpose, including Co−Cu/TiO_2_ for C_2_H_6_ and C_3_H_8_;^[7]^ single Au/red P,^[8]^ CdS/Cu−nanotube,^[9]^ Nafion−Pd−TiO_2_,^[10]^ Pt−graphene/TiO_2–*x*_,^[11]^ graphene−TiO_2_,^[12]^ and Au@Bi_12_O_17_Br_2_ for C_2_H_6_;^[13]^ CuPt_2_/TiO_2_ nanotube,^[14]^ Au−Pd/TiO_2_ {1 0 1},^[15]^ and TiON_1−(O vacancy)_ for C_2_H_6_ and C_2_H_4_;^[16]^ C/Cu_2_O nanorod,^[17]^ Cu/TiO_2_,^[18]^ Ag−C nanotube@TiO_2_,^[19]^ CuO/CuGaS_2_,^[20]^ Bi_2_S_3_@In_2_S_3_,^[21]^ FeCoS_2_,^[22]^ In_2.77_S_4_/porous polymer,^[23]^ N, S/Fe−MOF,^[24]^ and Cu^δ+^/CeO_2_−TiO_2_ for C_2_H_4_;^[25]^ forming the corresponding alcohol, aldehyde, and acid.[^3^, ^26^, ^27^, ^28^]

The number of reports on the photocatalytic synthesis of C_2_ and C_3_ HCs from CO_2_ has dramatically increased since 2019 (Table S1a, b, g, i, j, and n–s), yet a comprehensive understanding of the reaction pathways remains elusive, hindering precise control. This study reports the switchover of photocatalytic pathways from CO_2_ to C_1–3_ paraffin versus from CO to selective C_2_H_4_ and C_3_H_6_, using a Co^0^−ZrO_2_ catalyst (Supporting Information, *1.2. Major Framework of This Study*).

## Results and Discussion

**Figure 1:**
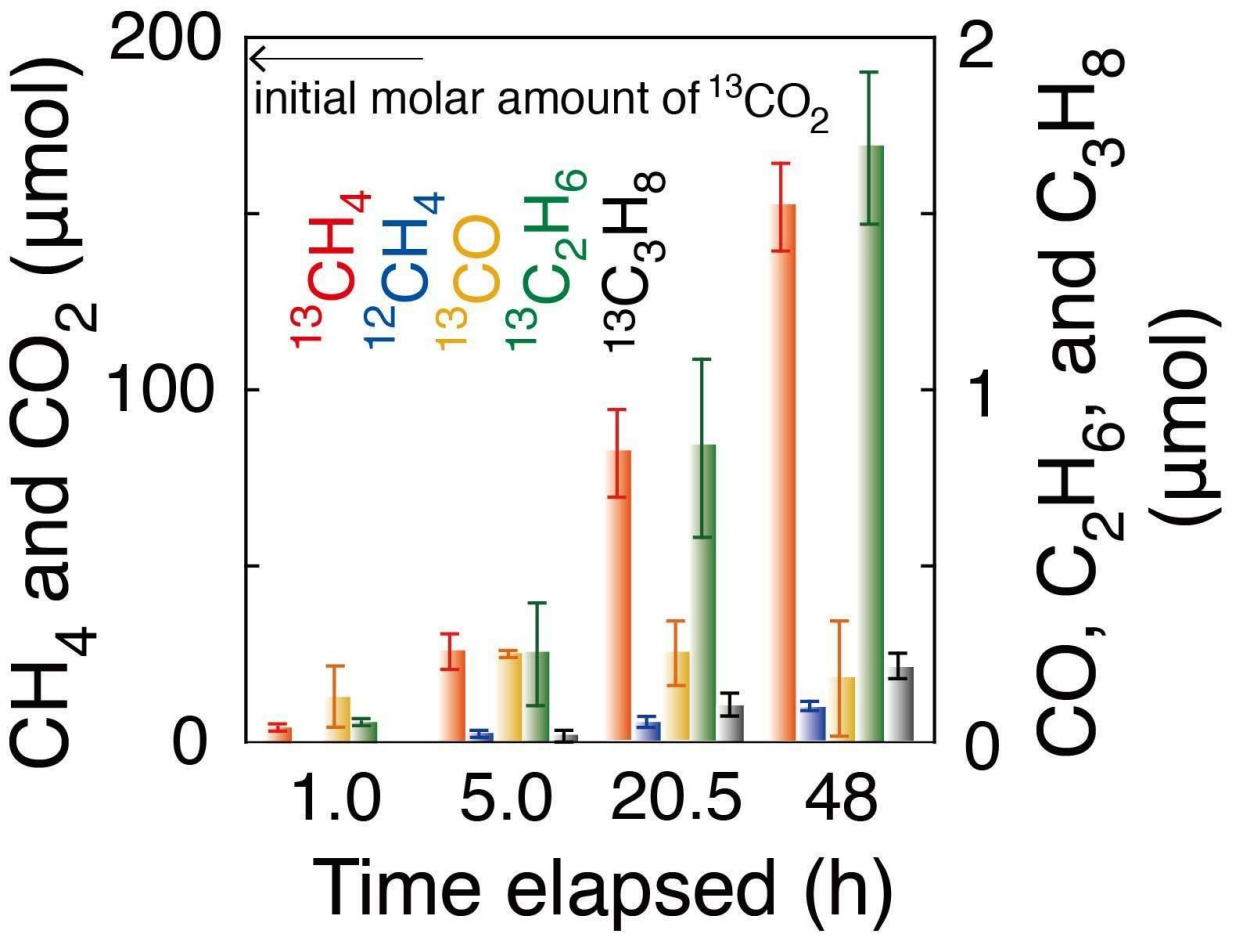
Time–course formation of photocatalytic ^13^CH_4_, ^12^CH_4_, ^13^CO, ^13^C_2_H_6_, and ^13^C_3_H_8_ during exposure to ^13^CO_2_ (2.3 kPa) and H_2_ (21.7 kPa) irradiated under UV/Visible light (142 mW cm^−2^) using Co (7.5 wt %)–ZrO_2_ (0.020 g) treated in H_2_ at 823 K. The error bars for each product were evaluated based on three factors described in SI, *3.1. Kinetic Results*.

Photocatalytic ^13^CO_2_ Reduction Using H_2_. The photocatalytic reduction tests of ^13^CO_2_ were first performed using ZrO_2_−823R, Co−ZrO_2_−823R (where 823 denotes the pretreatment temperature (K) of the photocatalysts with H_2_ and R represents reduced), and a Xe arc lamp guided through a quartz light conduit (142 mW cm^−2^; Table 1a–i). ZrO_2_ mostly reflected/scattered 94.5 %±0.3 % of light (300 nm<wavelength *λ*<2800 nm), while Co−ZrO_2_‐fresh, −723R, and −973R mostly absorbed 93.2 %±0.5 %, 93.2 %±0.3 %, and 97.2 %±0.2 % of light, respectively (SI, *2. Experimental Section*). In contrast to the sluggish formation of ^13^CO using ZrO_2_−823R (Table S3a), the utilization of Co−ZrO_2_−823R comprising 2.5–10 wt % of Co resulted in the predominant formation of ^13^CH_4_ accompanied by ^12^CH_4_, ^13^C_2_H_6_, ^13^CO, and ^13^C_3_H_8_ (Tables 1c and S3c–e and i). ^13^CO emerged as the primary product within 5 h of the reaction, followed by ^13^C_1–3_ paraffin formation (Figure 1). Among these catalysts, Co (7.5 wt %)–ZrO_2_−823R exhibited the highest total formation rate of HCs and CO (Tables 1c and S3e and Scheme S1A). This study confirmed a similar distribution of C_1–3_ paraffin distribution using Co−Cu/TiO_2_ (Table S1a)^[7]^ via monitoring of ^13^C‐isotopic time‐course dynamics.

**Table 1:** Kinetic Data for Photoconversion of CO_2_ or CO Using Co (7.5 wt %)–ZrO_2_ Photocatalysts Irradiated under UV/Visible Light.

					Formation rate (μmol h^−1^ g_cat_^−1^)							
Entry	C oxide	Reductant	*T* (K) under H_2_	Light intensity	^13^CO	^13^CH_4_	^12^CH_4_	^13^C_2_H_4_	^13^C_2_H_6_	^13^C_3_H_6_	^13^C_3_H_8_	^16^O_2_
a	^13^CO_2_ (2.3 kPa)	H_2_ (21.7 kPa)	–	142 mW per 1 cm^2^‐cat*^1^	0.016^[^*^3]^	<0.002		<0.002	<0.002	<0.002	<0.002	<0.002
b			723		0.29^[^*^3]^	1.1	0.14		0.011			
c			823		3.6^[^*^3]^	300	23		4.1		0.41	
d			973		11^[^*^3]^	190	8.9		3.4		0.25	
e		H_2_ (2.3 kPa)			40	52	3.3		1.1		0.082	
f		H_2_O (2.2 kPa)	823		2.6	16	3.4		0.20		0.014	
g	^13^CO (2.3 kPa)	H_2_ (2.3 kPa)			–	15	1.1	5.5^[^*^2]^	0.70		0.098	<0.002
h		H_2_ (21.7 kPa)	973			8.6	1.9	0.061	1.3	0.67	1.7	
i		H_2_ (2.3 kPa)				2.2	0.18	5.2^[^*^4]^	0.23	0.74^[^*^4]^	0.024	
					CO	CH_4_		^12^C_2_H_6_		^12^C_3_H_8_		O_2_
j^[^*^5]^	CO_2_ (95 kPa)	H_2_O (70 mL)	973	90.2 mW per flask^[^*^2]^	0.90	0.25		0.071		0.024		110
k^[^*^5]^			823	222 mW per flask^[^*^2]^	41	8.8		1.6		1.0		590
l^[^*^5]^			973		19	3.4		1.5		0.52		66

[*1] Using a quartz reactor (Chart S1). [*2] Using a Pyrex flask equipped with quartz window (Chart S2). [*3] Formation ceased within 1–4 h of reaction owing to the subsequent consecutive reactions. [*4] Selected consecutive reactions with the highest rates. [*5] Photocatalyst quantities were 20 mg each, except for entries j (9.0 mg), k (3.5 mg), and l (16 mg).

The performance of nonheated Co (7.5 wt %)–ZrO_2_ under H_2_ was even poorer than ZrO_2_ (Tables 1a and S3a). This aligns with previous findings on photocatalytic CO formation using CO_2_ and Co_3_O_4_ nanoparticles, which necessitated a Ru photosensitizer and triethanolamine.^[29]^ Next, the reduction temperature in H_2_ of the Co (7.5 wt %)–ZrO_2_ photocatalyst was varied between 723 and 973 K (Figure S2A–C and Table 1b–d). The major ^13^CH_4_ formation rate increased by 280 times with Co−ZrO_2_−823R compared to −723R. However, this rate dropped to 63 % with Co−ZrO_2_−973R compared to that using −823R, owing to the progressive reduction from Co_3_O_4_ to metallic Co^0^ and the possibly superior reactivity of the face‐centered cubic (fcc) Co^0^ surface versus hexagonal close‐packed (hcp) one^[30]^ (see the following *UV/Visible spectra, high‐resolution transmission electron microscopy (HR‐TEM), and X‐ray absorption near‐edge structure (XANES)* sections). More than 87 % of the formation rates were retained in repeated photocatalytic tests (Figure 1), provided that adsorbent balance on the Co^0^ surface was maintained (SI, *3.1. Kinetic Results*).

**Scheme 1:**
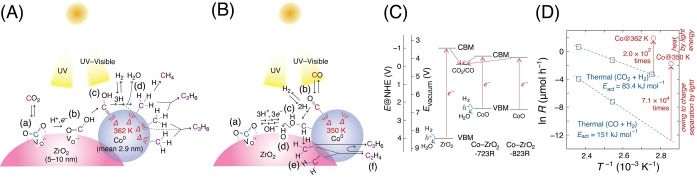
(A, B) Proposed Mechanisms for CO_2_ Photoreduction to C_1–3_ Paraffin (A) and CO Photoreduction to C_2,3_ Olefins (B); (C) the Energetics of First Half of Photocatalytic Reaction Steps from CO_2_ to CO and the Electron Flows; and (D) Comparisons of ^13^CO_2_ and ^13^CO Reduction Rates between Photocatalytic (circle, ○) versus Thermal (square, □) Processes Using Co−ZrO_2_−823R for ^13^CO_2_ and Co−ZrO_2_−973R for ^13^CO Reduction.

The obtained molar ratio of C_1–3_ paraffin (^13^CH_4_, ^12^CH_4_, ^13^C_2_H_6_, to ^13^C_3_H_8_) was almost constant at 100: 4.7–13: 0.98–1.8: 0–0.14 (Table 1b–d), regardless of the reduction temperature under H_2_. This consistency suggests a common reaction pathway, independent of the reduction temperature (Scheme 1A). The considerably slow dissociation of the first C−O bond in the CO_2_‐derived intermediate on O vacancy (Vo⋅⋅) sites at the ZrO_2_ surface (Scheme 1A–b, c) is likely rate‐limiting,^[31]^ thereby determining the overall rates. We previously reported the photocatalytic roles of Vo⋅⋅ sites on CO_2_ photoreduction.^[31]^ Subsequent steps from COH involve common progressive hydrogenation toward C_1–3_ paraffin over Co^0^ sites, resulting in a very similar paraffin ratio (Figure S2A–C and Scheme 1A–c, d; see the following *density functional theory* (*DFT) calculations* section).

Even when only visible light was utilized for irradiation, the C_1–3_ paraffin ratio remained consistent, suggesting a similar reaction pathway involving the utilization of Vo⋅⋅ sites at the ZrO_2_ surface (Table S3e, g, and h; SI, *3.1. Kinetic Results*).

Photocatalytic ^13^CO Reduction. The CO photoreductions were tested using the most active Co (7.5 wt %)–ZrO_2_−823R compared to the CO_2_ photoreduction reactions (Scheme S1A). When ^13^CO (2.3 kPa), H_2_ (2.3 kPa), and UV/Visible irradiation were used, ^13^C_2_H_4_ became the second major product (24 mol %) following ^13^CH_4_ (67 mol %; Figure S3 and Table 1g). ^13^C_2_H_4_ was the primary product, followed by secondary ^13^C_2_H_6_ evolution after 5 h of reaction, demonstrating a consecutive first‐order reaction kinetic model (Eq. S5 and Figure S4), with a CH_2_ intermediate, followed by C_2_H_4_, then C_2_H_6_ generations. The discrepancy between the amount of CO lost and the amount of products was mostly due to bidentate formate formation on ZrO_2_ surface^[32]^ (see *Reaction Mechanism* section and Figure S16) because formic acid and C_1–3_ alcohols were not found and negligible change of OH stretching vibration region in Fourier transform infrared (FTIR) spectra under the photocatalytic conditions. The molar ratio of C_1–3_ HC formation rates, ^13^CH_4_, ^12^CH_4_, ^13^C_2_H_4_, ^13^C_2_H_6_, to ^13^C_3_H_8_, was determined to 100: 7.2: 36: 4.6: 0.64 for photoreduction starting from ^13^CO (2.3 kPa) and H_2_ (2.3 kPa), differing from the values of 100: 7.7: 0: 1.4: 0.14 for photoreduction starting from ^13^CO_2_ (2.3 kPa) and H_2_ (21.7 kPa) (Table 1c and g). In the photocatalytic test using CO_2_ and H_2_, CO was negligibly identified as the intermediate, suggesting either its absence in the reaction pathway or the presence of a specific active site that selectively activates CO over CO_2_. While this aspect has been discussed for CO_2_ electroreduction,^[4]^ it has received limited attention in CO_2_ photoreduction.^[3]^

**Figure 2:**
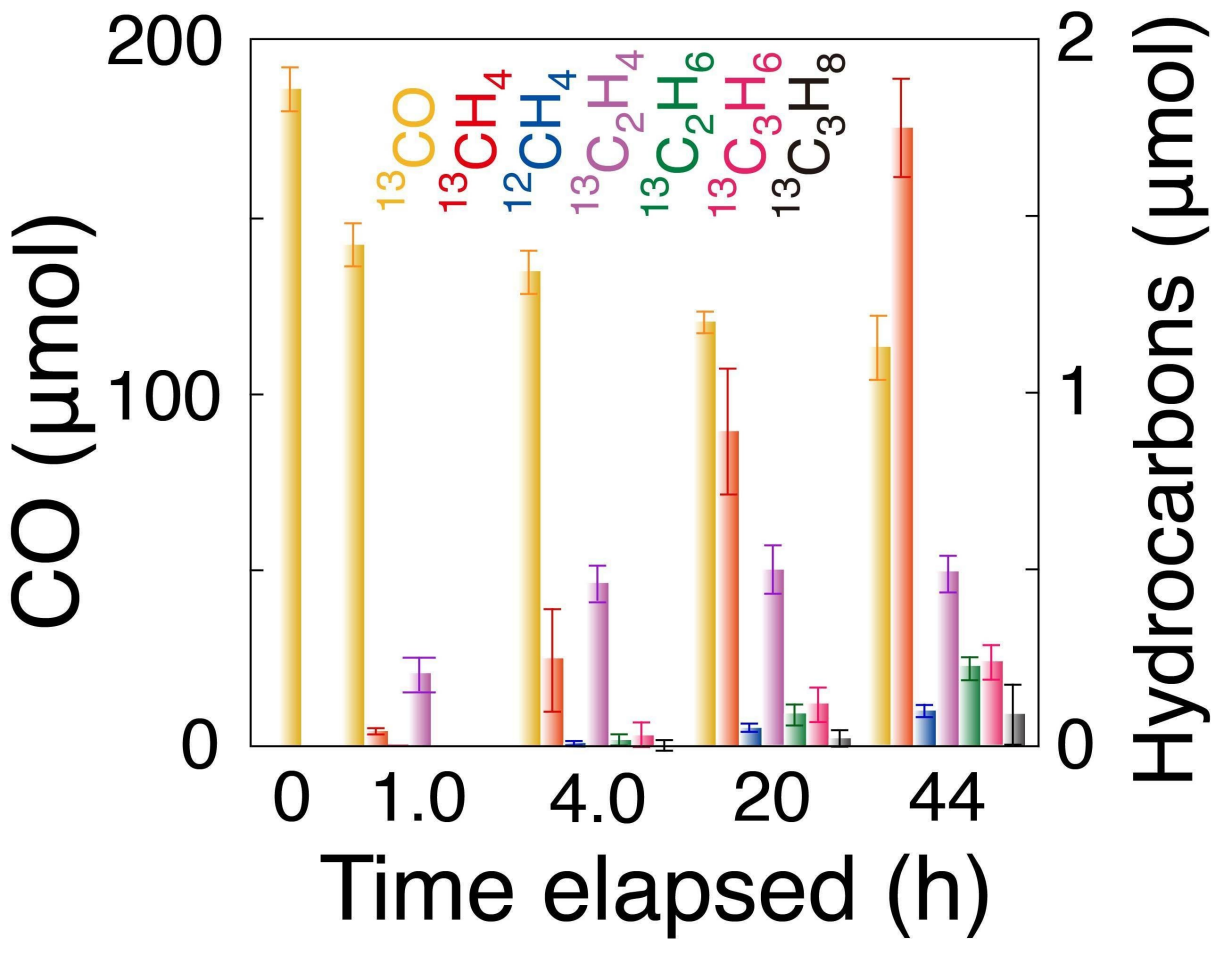
Time–course formation of photocatalytic ^13^CH_4_, ^12^CH_4_, ^13^C_2_H_4_, ^13^C_2_H_6_, ^13^C_3_H_6_, and ^13^C_3_H_8_ and the decrease of ^13^CO during exposure to ^13^CO (2.3 kPa) and H_2_ (2.3 kPa) irradiated under UV/Visible light (142 mW cm^−2^) using Co (7.5 wt %)–ZrO_2_ (0.020 g) treated in H_2_ at 973 K. The error bars for each product were evaluated based on three factors described in SI, *3.1. Kinetic Results*.

^12^CH_4_ originated from ^12^CO_2_ in the air, which chemisorbed onto Vo⋅⋅ sites at the ZrO_2_ surface.[^31^, ^32^, ^33^] This was confirmed by X‐ray photoelectron spectroscopy upon the introduction of CO_2_ and H_2_ to Co−ZrO_2_−823R (Figure S8A and B): the positive shift of Zr 3d peak indicating more Zr^4+^ population (+0.14 eV, panel A) shifted from Zr^3+^ associated with Vo⋅⋅ site and the growth of O (surface) peaks in the region 535.5–531.5 eV compared to O (lattice) at 530.1 eV in O 1s region (panel B).

Furthermore, using the Co (7.5 wt %)–ZrO_2_−973R photocatalyst, ^13^CO (2.3 kPa), H_2_ (2.3 kPa), and UV/Visible light (Figure 2) resulted in olefins as the major products: ^13^C_2_H_4_ (61 mol %) and ^13^C_3_H_6_ (8.6 mol %), rather than ^13^CH_4_ (25 mol %; Table 1i and Scheme S2B). The formation rate (5.2±0.5 μmol h^−1^ g_cat_^−1^) and selectivity (61 mol %) of ^13^C_2_H_4_ are comparable to those reported in 2023 (12–68 μmol h^−1^ g_cat_^−1^, 11–86 mol %; Table S1n–r).[^20^, ^21^, ^22^, ^23^, ^24^] However, the photocatalytic C_3_H_6_ formation (0.74 μmol h^−1^ g_cat_^−1^) from CO and/or CO_2_ is uncommon, with ^13^C_2_H_4_ being formed first, followed by ^13^C_2_H_6_, then ^13^C_3_H_6_ and ^13^C_3_H_8_ in consecutive order (Figure 2). Such consecutive reactions were well reproduced.

The ratio of ^12^CH_4_ among the total CH_4_ formed did not align with the impurity ratio of ^12^CO_2_ in the ^13^CO_2_ reagent used (1 %) but instead ranged from 4.5 % to 12 % during the tests using ^13^CO_2_ and H_2_ (Table S4b–e). This suggests that gas‐phase ^13^CO_2_ was in equilibrium with chemisorbed ^12^CO_2_ on Vo⋅⋅ sites at the ZrO_2_ surface from the air (Scheme 1A–a). When ^13^CO and H_2_ were used, the ratio of ^12^CH_4_ among the total CH_4_ formed was 6.7–18 % (Table S4g–i). Thus, gas‐phase ^13^CO was also in equilibrium with chemisorbed ^12^CO_2_ on Vo⋅⋅ sites via Eqs. 1 and 2.

(1)

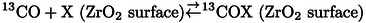



(2)

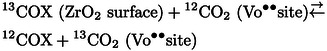




One possibility of such equilibrium is shown in Scheme 1B–a–c via HCOH species (X=2H in Eq. 1). Such photocatalytic reaction pathway is specific in contrast to thermal CO_2_ conversion, which predominantly occurs over the Co^0^ surface at temperatures higher than 425 K (SI, *3.1. Kinetic Results*).^[3]^

**Figure 3:**
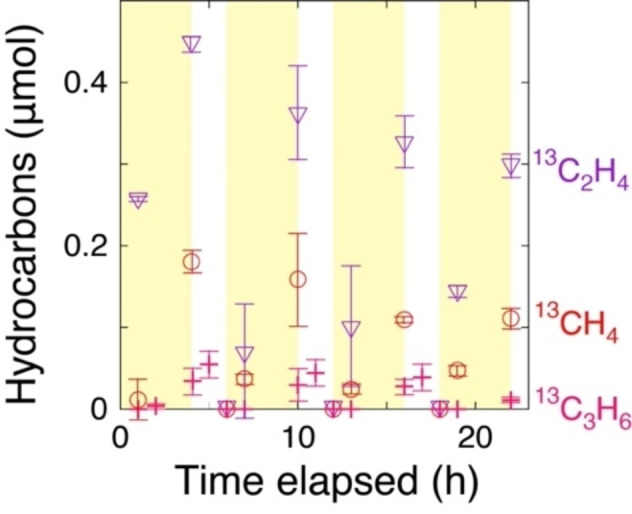
Time–course formation of photocatalytic ^13^CH_4_, ^13^C_2_H_4_, and ^13^C_3_H_6_ (i) during exposure to ^13^CO (2.3 kPa) and H_2_ (2.3 kPa) for 4 h, followed by (ii) 1 h of evaluation and (iii) subsequent exposure to ^13^CO (2.3 kPa) for 1 h using Co (7.5 wt %)–ZrO_2_−973R (0.020 g) irradiated under UV/Visible light (142 mW cm^−2^). The cycle of steps (i)–(iii) was repeated four times. The error bars for each product were evaluated based on three factors described in SI, *3.1. Kinetic Results*.

Switching Photocatalytic ^13^CO Reduction. We attempted to extract the first selective step of C_2_H_4_ and C_3_H_6_ formation in consecutive photocatalysis using ^13^CO, H_2_, and the optimal Co (7.5 wt %)–ZrO_2_−973R photocatalyst (Scheme S1B) by switching the reaction gases as follows: (i) under ^13^CO (2.3 kPa) and H_2_ (2.3 kPa) for 4 h, (ii) under vacuum for 1 h, then (iii) under ^13^CO (2.3 kPa) for 1 h. These steps were repeated (Figure 3). At step i of the first cycle, the molar ratio of olefin (^13^C_2_H_4_ and ^13^C_3_H_6_) formation was measured as 57 mol % after 4 h of reaction, similar to the initial stage of Figure 2.

The control key for the selective formation of C_2_H_4_ and C_3_H_6_ was the concentration of CH_2_ intermediate on the Co surface (Scheme 1B–e and Figure S4), which progressively decreased, resulting in the switching from C_2,3_ olefin to paraffin formation within 4 h of photoreaction. The HC intermediates on the Co surface were effectively removed under vacuum conditions (Scheme 1B–e and f), followed by ^13^CO adsorption during steps ii and iii (Figure 3).

In the second cycle, at step i, the total formation rate after 4 h of reaction was 84 mol % of that observed in the first cycle. The molar ratio of olefin formation was 57 mol %. Subsequently, in the third cycle, at step i, the total formation rate after 4 h of reaction decreased to 69 mol % of the first cycle, with the olefin formation molar ratio measured at 60 mol %. Furthermore, in the fourth cycle, at step i, the total formation rate after 4 h of reaction was 62 mol % of that observed in the first cycle, with the olefin formation molar ratio of 61 mol %.

The switching olefin photoformation was nicely reproduced in separated test under ^13^CO and H_2_, vacuum, then ^13^CO (Figure S5); olefin formation molar ratio gradually increased from 70 to 77, 83, and 87 mol % while total formation rate gradually decreased from 100 to 99, 68, and 55 mol %. Due to the nature of consecutive reaction, olefin selectivity critically depended on time exposed to ^13^CO, H_2_, and UV/Visible light (4–10 h). Upon repetition of the cycle, the ratio of ^13^C_2_H_4_ and ^13^C_3_H_6_ among all the HCs increased owing to the gradual accumulation of CH_2_ and/or C_2_H_4_ intermediate species (Scheme 1B–e) over the Co^0^ surface compared to CO and CH_3_ species, even after flushing under vacuum and exposure to ^13^CO between cycles (see following *FTIR spectroscopy* section). Thus, the first step of the consecutive photocatalytic CO reduction, i.e. C_2,3_ olefin formation, using the Co (7.5 wt %)–ZrO_2_−973R photocatalyst was successfully extracted.

Pressure Dependence of ^13^CO_2_/^13^CO Reduction and ^13^CO_2_ Uptake/Exchange. To provide insights into reaction mechanism, the pressure dependence of the reactant was considered. Using Co (7.5 wt %)–ZrO_2_−973R (Scheme S1B), ^13^CO_2_ (2.3 kPa), and H_2_ (2.3–21.7 kPa), the formation rate ratio of ^13^CH_4_, ^12^CH_4_, ^13^C_2_H_4_, ^13^C_2_H_6_, ^13^C_3_H_6_, to ^13^C_3_H_8_ was essentially constant: 100 : 4.7–6.4 : 0 : 1.8–2.2 : 0 : 0.13–0.16 (Table 1d and e), indicating no competition between CO_2_ and H_2_ for adsorption. In contrast, using CO (2.3 kPa) and H_2_ (2.3 kPa), the ratio was skewed toward olefin selectivity, measuring 100 : 8.6 : 243 : 11 : 34 : 1.1, which differs from the 100 : 23 : 0.71 : 15 : 7.8 : 20 observed with CO (2.3 kPa) and H_2_ (21.7 kPa; Table 1h and i). This suggests that the surface concentration of CH_2_ species critically depended on H concentration (Scheme 1B–e). Conversely, high H_2_ pressure decreased the ^13^CO formation rate using ^13^CO_2_ and ZrO_2_ (Table S3a and b), suggesting competitive adsorption of CO_2_ on Vo⋅⋅ sites and H on neighboring Zr sites.^[31]^

**Figure 4:**
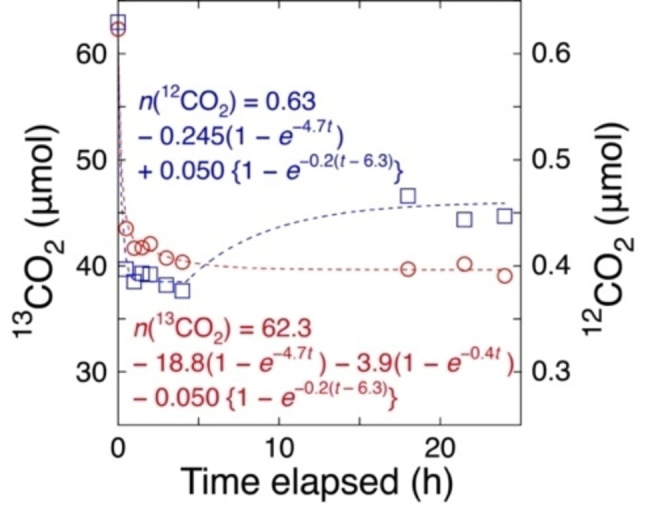
Time–course uptake and exchange reaction of ^13^CO_2_ (0.68 kPa) under UV/Visible light irradiation (142 mW cm^−2^) using Co (7.5 wt %)–ZrO_2_−823R and the fit equation curves based on first order kinetics. The amount of catalyst is 0.020 g.

Then, the CO_2_ adsorption sites were investigated using ^13^CO_2_ uptake and exchange reactions with Co (7.5 wt %)–ZrO_2_−823R (Scheme S1A) and UV/Visible light irradiation (Figure 4). At a rate constant of 4.7 h^−1^, the initial rapid uptake of ^13^CO_2_ and 1.0 % impurity ^12^CO_2_ (in total 19.0 μmol) were attributed to physisorption on the ZrO_2_ surface. The additional uptake (9.1 μmol) of CO_2_ compared to the 9.9 μmol‐CO_2_ using the same amount (20 mg) of undoped ZrO_2_ catalyst (Table S5a and b)^[32]^ corresponded to 36 mol % of the Co‐site in the Co (7.5 wt %)−ZrO_2_−823R catalyst. Thus, rapid CO_2_ adsorption on Co^0^ was implausible, and the extra uptake was attributed to the formation of Co carbonate resulting from the reaction of CoO with CO_2_.

Subsequent ^13^CO_2_ uptake on the Co (7.5 wt %)–ZrO_2_−823R was also physisorption at a rate constant of 0.4 h^−1^ (Figure 4 and Table S5b), suggesting adsorption at the interface sites. Conversely, the much slower ^13^CO_2_/^12^CO_2_ exchange reaction, with a rate constant of 0.2 h^−1^, was attributed to the chemisorption site, likely owing to the presence of Vo⋅⋅ sites. In this mechanism, the ^12^CO_2_ adsorbed from the air is exchanged with gas‐phase ^13^CO_2_ (Scheme 1A–a). The concentration was calculated to be one vacancy per a ZrO_2_ surface area of 61 nm^2^, in consistent with the total amount of ^12^CH_4_ formed using N_2_, H_2_, and UV/Visible light irradiation (Table S3f and SI, *3.1. Kinetic Results*).

Electronic Characterizations. Diffuse‐reflectance UV/Visible spectra were measured to monitor effective electrons by changing the reduction temperature of photocatalysts. In addition to the absorption edge observed at 248 nm for ZrO_2_ (Figure S6A–a), two peaks appeared at 387 and 687 nm for the fresh Co (7.5 wt %)–ZrO_2_ (spectrum b), attributed to the charge transfer from O^2−^ to Co^2+^ and O^2−^ to Co^3+^, respectively, in Co_3_O_4_.[^34^, ^35^] For Co (7.5 wt %)–ZrO_2_−723R, a single peak appeared at 612 nm owing to the charge transfer from O^2−^ to Co^2+^ in CoO (spectrum c).^[35]^ Based on the fitting of these spectra to the equation proposed by Davis and Mott,^[36]^

(3)

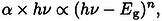




where *h* is Planck constant, *ν* is the frequency of light, and *α* is a constant, band gap *E*_g_ values of 2.4 and 2.9 eV were obtained for allowed direct transition (*n*=1/2)^[35]^ in Co_3_O_4_ and CoO nanoparticles over ZrO_2_ (Figure S7A and B and Scheme 1C), respectively.

The absorption in the range of 250–800 nm progressively increased with the elevation of the reduction temperature to 823 K and then to 973 K (Figure S6A–d and e), suggesting a complete reduction to metallic Co^0^ at 973 K, in accordance with the findings in the following *XANES/extended X‐ray absorption fine structure (EXAFS)* sections. However, the peak observed at 612 nm attributable to CoO (15 mol %) (intensity ~0.05) was not well resolved, as it overlapped with the absorption by Co^0^ (85 mol %, intensity 2–3) for Co (7.5 wt %)–ZrO_2_−823 R (see *XANES* section).

The energetics of the Co−ZrO_2_ photocatalysts are summarized in Scheme 1C. Concerning the valence band maximum (VBM) of ZrO_2_ (4.0 V vs. normal hydrogen electrode, NHE), the band gap value was 5.0 eV based on the UV/Visible spectrum (Figure S6A–a), while the conduction band minimum (CBM) was at −1.0 V vs. NHE. The VBM values of CoO in Co (7.5 wt %)–ZrO_2_−723 R and −823R photocatalysts were calculated to 2.3 and 2.4 V vs. NHE (Scheme 1C), respectively (Figure S8C and SI, *3.2. Characterizations*).

Based on the band gap values for CoO (2.9 eV) shown in Figure S7B, its CBMs were at −0.6 and −0.5 V (SI, *3.2. Characterizations*). Thus, UV and/or visible light‐excited electrons to the CB of both ZrO_2_ and CoO could thermodynamically reduce CO_2_ to CO (−0.11 V; Scheme 1C). Thus, the presence of CoO adjacent to Co^0^ nanoparticles can boost CO_2_ photoreduction by leveraging visible light.

The efficiency of charge separation in photocatalysts by light was evaluated by fluorescence spectroscopy (Scheme S1C). The fluorescence peak intensity associated with the near band‐edge and the midgap trap states was suppressed to one‐tenth to one‐fifth for Co (7.5 wt %)–ZrO_2_ compared to corresponding peaks for ZrO_2_ owing to the charge transfer of light‐excited electrons to Co_3_O_4_, CoO, or Co^0^. The associated peaks in excitation spectra were substantially suppressed owing to the charge transfer effect to Co species in accordance with fluorescence spectra (Figure S6B and C; SI, *3.2. Characterizations*) and in‐profile CO_2_ photoreduction tests suggested an auxiliary role of vacancy/impurity level of ZrO_2_ (SI, *3.1. Kinetic Results*), suggesting the utilization of the charges for photocatalysis.[^1^, ^2^]

**Figure 5:**
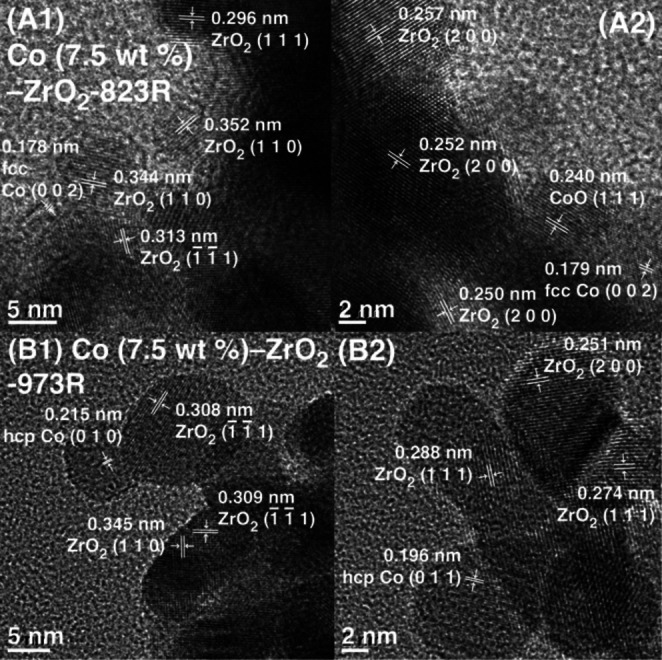
HR‐TEM images of Co (7.5 wt %)–ZrO_2_−823R (A1, A2) and Co (7.5 wt %)–ZrO_2_−973R photocatalysts (B1, B2). Lattice intervals for monoclinic ZrO_2_ (all panels), CoO (A2), fcc Co (A1, A2), and hcp Co (B1, B2) were also drawn.

Structural Characterizations. The morphology of the Co (7.5 wt %)–ZrO_2_−723R (Figure S9), −823R, and −973R samples (Figure 5) was observed using HR‐TEM to provide structural information on nano level and the changes of especially Co sites owing to reduction temperature (Scheme S1C). The monoclinic phase of ZrO_2_ crystals with 5–10 nm (Scheme 1) in size was preferably observed, exhibiting lattice fringes with the intervals of 0.274–0.296, 0.344–0.352, 0.246–0.257, 0.240, 0.264–0.267, and 0.308–0.313 nm corresponding to ZrO_2_ (1 1 1), (1 1 0), (2 0 0), (0 2 1), (0 0 2), and ($\bar{1}\;\bar{1}\;1$ ) (theoretical values 0.285, 0.365, 0.255, 0.234, 0.264, and 0.318 nm),[^32^, ^33^] respectively, for these three samples.

In contrast to Co (7.5 wt %)–ZrO_2_−723R, where cubic CoO was observed (1 1 1) lattice interval=0.247 nm, theoretical=0.245 nm; (0 0 2) lattice interval=0.205–0.206 nm, theoretical=0.212 nm)^[37]^ (Figure S9), for Co (7.5 wt %)–ZrO_2_−823R, fcc Co nanoparticles were observed (0 0 2) lattice interval=0.178–0.179 nm, theoretical=0.177 nm)^[37]^ in the proximity to CoO nanoparticles comprising (1 1 1) lattice with an interval of 0.240 nm,^[38]^ as shown in Figure 5A2. Furthermore, for Co (7.5 wt %)–ZrO_2_−973R, hcp Co nanoparticles were frequently observed with intervals of 0.215 and 0.196 nm corresponding to the (0 1 0) and (0 1 1) lattices (Figure 5B1, 2; theoretical=0.217 and 0.192 nm),^[39]^ respectively, while no CoO phase was observed. A few nanometer‐sized Co nanoparticles could exhibit fcc packing owing to the reduction at 823 K, while many Co crystals (>5 nm) grown at 973 K transformed into a stable hcp phase. The HR‐TEM results were well consistent with the X‐ray diffraction (Figure S10A), which showed diffractions from monoclinic ZrO_2_. However, only very weak traces of the shoulder owing to hcp Co (0 1 0) and (0 0 2) were identified (Figure S10B) because of their small size of a few nanometers.

Co K‐edge XANES spectra confirmed the speciation of Co: Co_3_O_4_, CoO, and Co^0^ metal for fresh Co (7.5 wt %)–ZrO_2_, Co (7.5 wt %)–ZrO_2_−723R, and −973R, respectively (Figure S11A and SI, *3.2. Characterizations*), consistent with UV/Visible absorption spectroscopy (Figure S6A). For Co (7.5 wt %)–ZrO_2_−823R, the Co site comprised a mixture of CoO (15 %) and Co^0^ (85 %) (Figure S11B). The CoO amount based on rapid CO_2_ physisorption (extra 9.1 μmol of CO_2_ corresponding to CoO 36 %; Table S5a and b) was greater than 15 %, evaluated through XANES, probably because slower CO_2_ physisorption at the interface between Co and ZrO_2_ surface was also included.

In the comparison between Co state and photocatalytic CO_2_ reduction, while the fresh Co (7.5 wt %)–ZrO_2_ and Co (7.5 wt %)−ZrO_2_−723R photocatalysts comprising Co_3_O_4_ and CoO, respectively (Figure S11A), exhibited poor activity for CO_2_ photocatalytic reduction (Tables 1a and b and Figure S2A), Co (7.5 wt %)–ZrO_2_−823R and −973R comprising metallic Co (Figure S11A) were the most and the second most active in the CO_2_ photocatalytic reduction (Scheme S1A and B, Table 1c and d, and Figures 1 and S2 C), respectively. Thus, metallic Co sites played an essential catalytic role combined with Vo⋅⋅ sites at ZrO_2_ surface[^31^, ^32^, ^33^] as dual active sites, similar to our previous reports for Ni−ZrO_2_ by experiments^[32]^ and DFT calculations.^[31]^ Three plausible reasons can be listed: (i) fcc Co^0^ surface was more conducive to dissociating COH species than hcp one,^[30]^ (ii) CoO could work as a promoter in contact with Co^0^ (Figure 5A2) owing to visible light absorption and charge separation with an *E*_g_ value of 2.9 eV (Figures S6A–c and S7B and Scheme 1C), and (iii) Co^0^ particle size may increase at 973 K. However, the major reason cannot be identified because these potential changes occurred simultaneously at 973 K, and the Co^0^ particle size mixed with CoO at 823 K was difficult to determine via EXAFS and HR‐TEM (Figure 5A).

Photothermal Monitoring and the Control Thermal ^13^CO_2_/^13^CO Reduction. Related to the latter reaction steps of CO_2_ photoreduction over Co sites followed by CO_2_ activation at Vo⋅⋅ sites on ZrO_2_ surface, the local electronic and geometric structures associated with the thermal behavior of active metallic Co sites essential for CO_2_ photoreduction were monitored via Co K‐edge EXAFS spectroscopy. The analysis was conducted under UV/Visible light irradiation using CO_2_ (2.3 kPa), H_2_ (21.7 kPa), and Co (7.5 wt %)–ZrO_2_−973R. To precisely analyze Co^0^ sites not mixed with CoO, the second‐best catalyst was chosen (Table 1d and Scheme S1B). In the Fourier transform of EXAFS before UV/Visible light irradiation, the curve‐fit analysis revealed an intense peak observed at 0.21 nm (uncorrected for phase shift; Figure S12). This peak demonstrated a Co−Co interatomic distance (*R*) of 0.249 nm with an associated coordination number (*N*) of 9.8. Furthermore, the *N* and *R* values did not change considerably during the photocatalytic reaction (Figure S13A and B). The *N* value corresponds to a particle size of 2.9 nm, assuming a spherical fcc nanoparticle model with a surface dispersion (*D*) was 0.43.^[40]^

The Debye–Waller factor (*σ*) was calculated using the correlated Debye model[^41^, ^42^] for bulk and surface Co sites (vertical motion versus surface),[^32^, ^33^] considering the Debye temperature for bulk *θ*_D(Bulk)_ (445 K)^[43]^ and surface *θ*_D(Surface,⊥)_ for the vertical motion of freedom (211 K;^[44]^ Figure S1). We approximated the mean temperature of Co nanoparticle (*T*_nanoparticle_) as the arithmetic mean value, considering *θ*_D(Surface,⊥)_ weighted using 1/2 ⋅ 1/3 *D* for vertical translational motion at a free hemisphere surface and *θ*_D(Bulk)_ weighted using (1−*D*)+(1/2)*D*+(1/2 ⋅ 2/3)*D* for the bulk site, nonfree hemisphere in contact with ZrO_2_ surface, and horizontal translational motion at a free hemisphere surface.
(4)

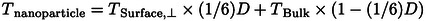




The *σ* value for samples (*σ*_sample_) was calculated using Eq. 5, taking the contribution of structural disorder (*σ*_disorder_) and the difference of the *σ*_sample_ value from the Co metal foil (*σ*_XDAP_) into account:
(5)

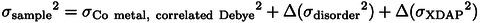




At 296 K, *σ*_sample_ and *σ*_Co metal, correlated Debye_ values were 0.007 (28) and 0.007 (05) nm, respectively, for the nanoparticle model above based on the correlated Debye model (Figure S1) and Eq. 4. The *σ*_XDAP_ value was given as −0.000 (32) nm. Thus, *σ*_disorder_ was evaluated as 0.001 (82) nm using Eq. 5.

**Figure 6:**
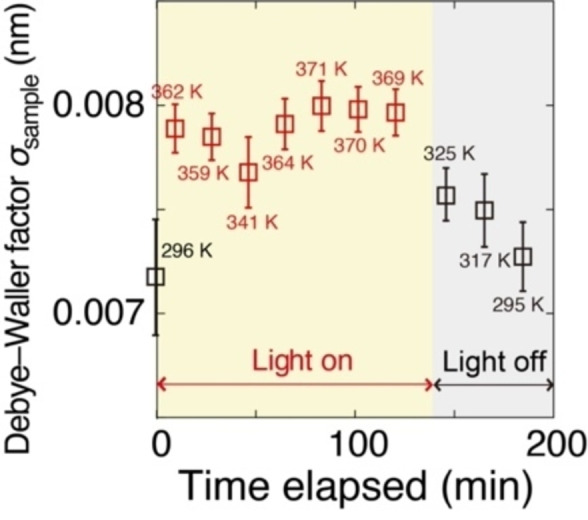
Time course of the Debye–Waller factor *σ* derived from the Co K‐edge EXAFS analysis and the determined temperature of Co nanoparticles in Co (7.5 wt %)–ZrO_2_−973R using CO_2_ (2.3 kPa), H_2_ (21.7 kPa), and UV/Visible light irradiation (142 mW cm^−2^), based on the correlated Debye model. The error bars were drawn based on data reproducibility in three runs and the fit errors.

Then, the temperature of the Co site was inversely evaluated based on the *σ*_sample_ value obtained from Eq. 5 and the arithmetic mean value based on Eq. 4 (Figure 6). This specific approach directly monitored the local Co site temperature.[^32^, ^33^] The Co sites were initially at 296 K before exposure to UV/Visible light. Upon irradiation, the temperature of the Co sites quickly increased to 362±21 K, gradually decreasing once the light was turned off after 138 min. The gradual drop in temperature post‐illumination cessation (Figure 6) is probably attributed to the presence of a minor CoO layer between the Co^0^ nanoparticle and the ZrO_2_ surface, which slows heat dissipation.

Compared with the critical change of *σ*_sample_ values triggered by UV/Visible light irradiation (Figure 6), the *N*(Co−Co) and *R*(Co−Co) values negligibly varied during UV/Visible light exposure and cessation, except for rapid initial quick changes upon the light activation (Figure S13). This initial change may result from the oxidation of minor Co^II^ sites by the effect of CO_2_ (Figure S11B), which were subsequently reduced under H_2_ and UV/Visible light. The reduced state of Co^II^ maintained a dynamic equilibrium under these conditions, with *N* values gradually decreasing upon light deactivation (Figure S13A).

The CH_4_ formation rate reached 196 μmol h^−1^ g_cat_^−1^ (Table 1d) under UV/Visible light irradiation leading to a temperature rise of 11.9 K within 10 min thermodynamically. However, this increase was smaller than the initial observed increase of 66 K within 10 min (Figure 6; SI, *3.2. Characterizations*). Thus, the temperature elevation in Figure 6 stemmed from the transformation of light energy into heat at the Co^0^ surface, quickly reaching the heat equilibrium and dissipating into the reactor/EXAFS cell.

Consistent with this evaluation of warming of Co^0^ sites by light under CO_2_, the time course of Fourier transform (Figure S14), *σ* value, and Co^0^ site temperature under CO, H_2_, Co (7.5 wt %)–ZrO_2_−973R (Scheme S1B), and UV/Visible light irradiation (Figure S15) reached 350±8 K and behaved very similarly to Figure 6, irrelevant to reactants.

To identify the origin of photocatalytic CO reduction in this study, control thermal reaction tests were performed using Co (7.5 wt %)–ZrO_2_−973R, ^13^CO, and H_2_ at a reaction temperature between 363 and 423 K (Table S6B). The total formation rate at 350 K was lower by a factor of 71 000 compared to that under UV/Visible irradiation (Scheme 1D and Table 1i), wherein the Co nanoparticles reached 350 K during the CO photoreduction test (Figure S15). This indicates that simple thermal catalysis did not occur solely owing to heat transformed from UV/Visible light energy. Similarly, the total formation rate at 362 K using Co (7.5 wt %)–ZrO_2_−823R as evaluated for Co (7.5 wt %)–ZrO_2_−973R (Figure 6), ^13^CO_2_, and H_2_ was lower by 200 times compared to that under UV/Visible irradiation (Table S6A and Scheme 1D). The C_2,3_ HC formation was especially suppressed in the thermal reaction, suggesting different reaction pathways under UV/Visible light irradiation (SI, *3.1. Kinetic Results*). The higher apparent activation energy (*E*_act_) from CO (151 kJ mol^−1^) compared to that from CO_2_ (83.4 kJ mol^−1^) contradicts the possibility of higher *E*_act_ from CO_2_ to CO that then follow common pathway. Instead, the fact suggests that CO followed a different thermal pathway from that from CO_2_, related to the difference of photocatalytic pathways between Scheme 1A and B.

**Figure 7:**
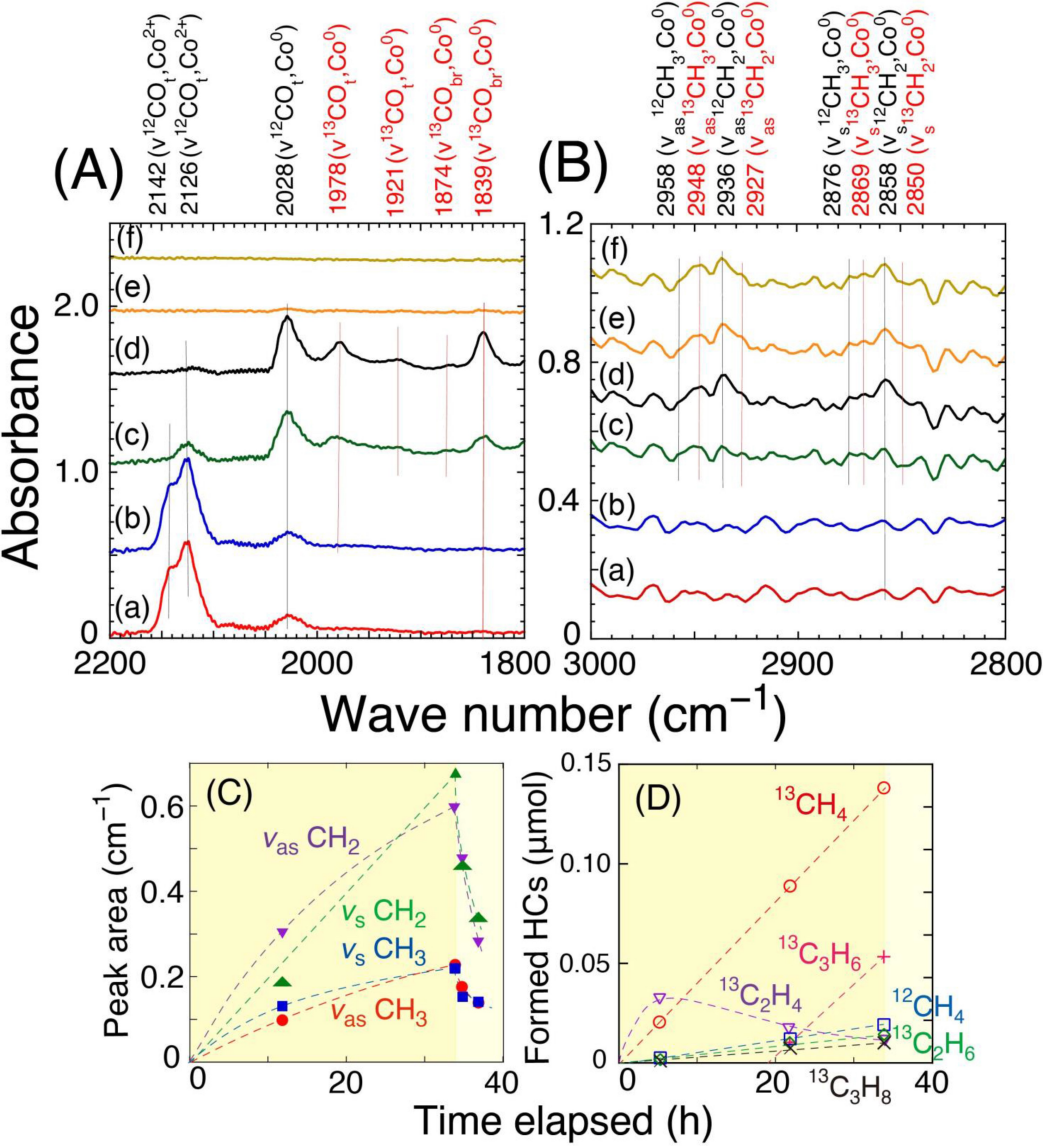
FTIR monitoring of reaction intermediates using the Co (7.5 wt %)–ZrO_2_–973R photocatalyst (71 mg) in the (A) carbonyl and (B) C−H stretching vibration regions and the peak assignments. The reaction conditions were as follows: (a) under ^13^CO (2.3 kPa) and H_2_ (2.3 kPa) in the dark for 2 h, (b–d) under ^13^CO (2.3 kPa), H_2_ (2.3 kPa), and UV/Visible light irradiation at 0 h (b), 12 h (c), and 34 h (d), and (e, f) under vacuum and UV/Visible light irradiation at 0 h (e) and 2 h (f). (C) The time course of CH_2_ and CH_3_ stretching vibration peak intensity (including convolution of ^13^C and ^12^C peaks) by FTIR and (D) the corresponding time course of photocatalytic products monitored using GC−MS.

Reaction Mechanism. FTIR spectroscopy investigated the specific photocatalytic reaction mechanism, starting from the conversion of CO to C_2_H_4_ and C_3_H_6_ using the optimal Co (7.5 wt %)–ZrO_2_−973R photocatalyst (Scheme S1B). Analysis of the ^13^C/^12^C isotopic distribution of the photocatalytic products, generated from ^13^CO_2_ and ^13^CO as starting materials (Table S4b–i) and ^13^CO_2_ exchange reaction (Figure 4), revealed that a part of the ^12^CO_2_ initially adsorbed from the air onto Vo⋅⋅ sites at the ZrO_2_ surface (one per the area of 61 nm^2^) remained after pretreatment under H_2_. Subsequently, this ^12^CO_2_ was then incorporated into the reaction pathway toward HCs (Scheme 1B–a). Migration of ^12^CO_2_ from Vo⋅⋅ sites to the surface of Co^0^ nanoparticles and its reaction with the surface (Eq. 6) demonstrated by the presence of ^12^CO stretching vibration (*ν* ^12^CO) peaks observed at 2142 and 2126 cm^−1^ (Figure 7A‐a) under ^13^CO and H_2_:
(6)

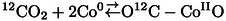




A minor part of the CO formed on the Co^II^ site in Eq. 6 further moved laterally (Figure 5A2) to the Co^0^ site (*ν* ^12^CO 2028 cm^−1^, Figure 7A–a). Because ^13^CO was not in direct equilibrium with ^12^CO_2_ at the Vo⋅⋅ sites, species derived from ^13^CO were not observed at 0 h under ^13^CO and H_2_ (Scheme 1B and Figure 7A–a and B–a).

When the UV/Visible light irradiation started, leading to a gradual decrease in the *ν* ^12^CO peak intensity on Co^II^ sites, the corresponding *ν* ^12^CO peak on the Co^0^ site observed at 2028 cm^−1^ progressively grew (Figure 7A–b and c). It was followed by a progressive increase in the isotopically‐labeled terminal and bridging *ν* ^13^CO peaks observed at 1978, 1921, 1874, and 1839 cm^−1^ (Figure 7A–c and d),^[45]^ demonstrating that the ^12^CO species derived from ^12^CO_2_ adsorbed at Vo⋅⋅ site when the light was turned on (0 h) were gradually replaced by gas phase ^13^CO (0–12 h), with ^13^CO on Co^0^ sites becoming the major species at 34 h under light (Figure 7A–d and Scheme 1B–b).

However, the isotopic ratio of the *ν* ^12^CO/*ν* ^13^CO peak intensity in FTIR (Figure 7A–b and c) and the ratios of ^12^C_2_H_4_/(^12^C_2_H_4_+^13^C_2_H_4_) and ^12^CH_4_/(^12^CH_4_+^13^CH_4_) (7.9 mol %, Tables 1i and S4i; Figure 7A–c and d) obtained from gas chromatography–mass spectrometry (GC−MS) were significantly greater than ^12^CO ratio in reagent (1 %). This discrepancy can be explained if the ^13^CO adsorbed on Co^0^ sites was in equilibrium with ^12^CO_2_ on Vo⋅⋅ site at ZrO_2_ surface (Scheme 1B–a; Eq. 6 and Figure 7A–a–d), and the CO adsorbed on Co^0^ sites should be the intermediate species toward HCs (Scheme 1B–b).

Synchronized with the growth of the *ν* ^12^CO peak (Figure 7A‐b–d), the antisymmetric and symmetric vibration of ^12^CH_2_ (*ν*_as_^12^CH_2_ and *ν*_s_^12^CH_2_) peaks increased at 2936 and 2858 cm^−1^, respectively, accompanied by weak vibration peaks for ^12^CH_3_ (*ν*_as_^12^CH_3_ 2958 cm^−1^; *ν*_s_^12^CH_3_ 2876 cm^−1^) under ^13^CO, H_2_, and UV/Visible light (Figure 7B–c). Then, following the isotopic replacement by multiple *ν* ^13^CO peaks (Figure 7A–b–d), the CH_2_/CH_3_ vibrational pairs of *ν*_as_^12^CH_*n*_ and *ν*_s_^12^CH_*n*_ (*n*=2, 3) progressively shifted to the corresponding ^13^C‐isotopic pairs of *ν*_as_^13^CH_*n*_ and *ν*_s_^13^CH_*n*_ (2927 and 2850 cm^−1^ when *n*=2 and 2948 and 2869 cm^−1^ when *n*=3, respectively, Figure 7B–d). Although the stretching vibration peak intensity ratio of CH_2_/CH_3_ in FTIR was almost constant at 3 : 1 during the 34‐hour photocatalytic test (Figure 7C), the major product obtained using GC−MS transformed consecutively transformed from ^13^C_2_H_4_ to ^13^CH_4_ (Figure 7D). This indicates that the ratio of CH_*x*_ to H adsorbed at a specific local site, e.g., the interface site between Co^0^ and the ZrO_2_ surface, determined the product selectivity (Scheme 1B–b–e). In contrast, the ^12^CO_3_ at the ZrO_2_ surface was replaced by ^13^CO_3_ species (1513 and 1308 cm^−1^), while bidentate ^13^C‐formate at the ZrO_2_ surface gradually increased (1523 and 1344 cm^−1^)^[32]^ until 34 h of reaction under ^13^CO, H_2_, and UV/Visible light (Figure S16), both irrelevant to HC generation over the Co^0^ surface.

Then, the Co (7.5 wt %)–ZrO_2_−973R photocatalyst subjected to vacuum and the UV/Visible light irradiation. In contrast to the quick disappearance of all the weakly adsorbed CO peaks (Figure 7A–e and f), approximately half of the reaction intermediate CH_2_ and CH_3_ species remained at 2 h under vacuum (Figure 7B–e, f and C), consistent with the HC preference using this photocatalyst.

**Scheme 2:**
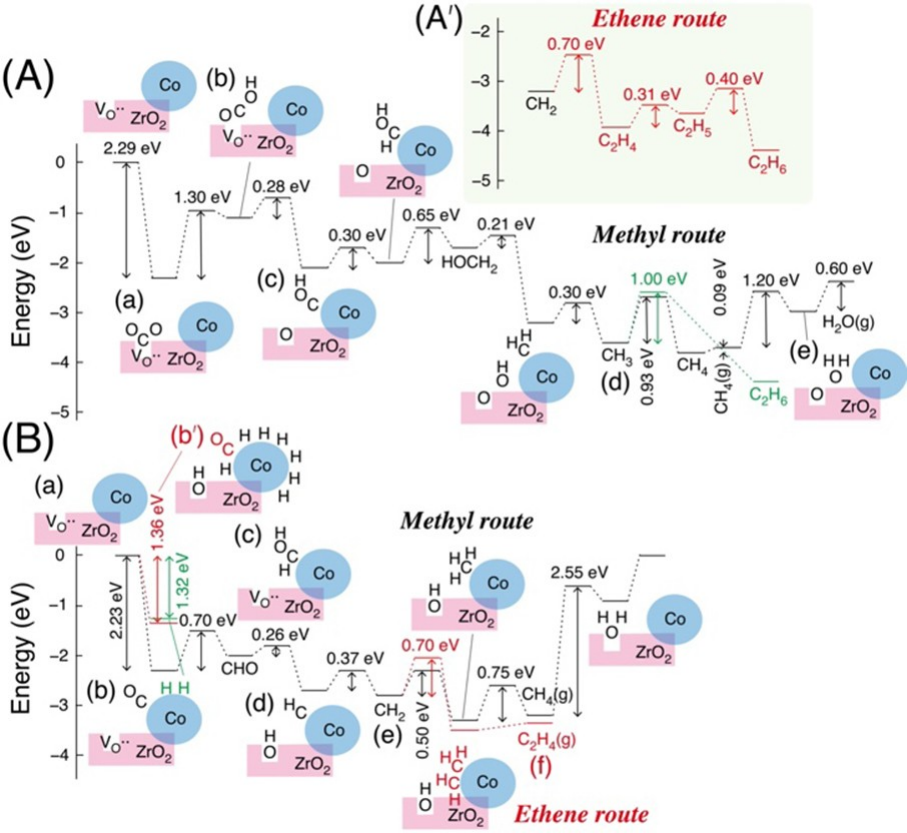
Energy Diagram over Monoclinic ZrO_2_ (1 1 1) Surface Combined with Co_19_ Cluster Exposing hcp (0 1 0) Surface Calculated (A) under CO_2_ and H_2_ and (B) under CO and H_2_. Ethene Route Was Also Drawn in (A′) and (B) Compared to Methyl Route. Three‐Dimensional Illustration of Each Species Is Presented in Scheme S2.

Spin‐polarized periodic DFT+*U* calculations were conducted using the Vienna Ab initio Simulation Package code version 6.2.1^[46]^ (SI, *2. Experimental Section*). CO_2_ was adsorbed at the Vo⋅⋅ site (Scheme 1A–a and Figure S8A and B).^[31]^ When H was provided, the adsorbed CO_2_ transformed to OCOH with an *E*_act_ of 1.3 eV (Scheme 2A–b). If the Vo⋅⋅ site neighbored a Co^0^ site, the terminal O atom of the OCOH group occupied the Vo⋅⋅ site (species b), and the COH moiety hopped onto the metallic Co^0^ atom with an *E*_act_ of 0.28 eV (species c). A very similar reaction mechanism was also proposed for Pd−ZrO_2_.^[47]^ The population of Vo⋅⋅ sites adjacent to the Co^0^ nanoparticle was anticipated to be relatively higher compared to the mean population of one Vo⋅⋅ per ZrO_2_ surface area of 61 nm^2^ (Table S5b). This is because the Fermi level of Co (work function 5.0 eV)^[48]^ is lower than the energy level of Vo⋅⋅ in ZrO_2_,^[31]^ enabling electron acceptance,^[49]^ thereby facilitating the generation of Vo⋅⋅ sites neighboring to Co^0^ nanoparticles.

Throughout the pathway to CH_4_, the hydrogenation to OCOH required the highest *E*_act_ of 1.3 eV, followed by the *E*_act_ of 0.93 eV for CH_3_ hydrogenation, and 1.2 eV for OH hydrogenation by the elimination at the interface (Scheme 2A–a, b, d, and e, *Methyl route*), thereby supporting the preferential formation of CH_4_ from CO_2_ (Table 1b–e). The regeneration energy of the Vo⋅⋅ site can be minimized to 1.7 eV by replacing H_2_O with CO_2_ at the Vo⋅⋅ site.^[31]^ C_2_H_6_ was formed via the *Ethene route* (Scheme 2A′) as a byproduct, preferably via C_2_H_4_ (*E*_act_ of 0.70 eV) rather than the coupling of two CH_3_ (*E*_act_ of 1.0 eV; Scheme 2A–d, Figure S4, and Eq. S1), different from the situation using Ni−ZrO_2_ photocatalyst, where CH_4_ was exclusively formed.^[31]^ The dissociation of HOCH_2_ into hydroxy and CH_2_ at the interface was also suggested (Scheme 2A), in contrast to considerably stable HOCH and dissociation on the Ni^0^ surface.^[31]^

The reaction route of CO photoreduction, especially to C_2_H_4_, was also investigated by DFT calculations. In contrast to CO_2_ adsorption at the Vo⋅⋅ site of the ZrO_2_ surface,[^31^, ^32^, ^33^] CO is adsorbed on the Co^0^ site (the adsorption energy (*E*_ads_) of 2.23 eV; Schemes 1B–b and 2B–b), followed by the formation of CHO and HCOH species at the interface of the ZrO_2_ surface and Co^0^ (Schemes 1B–c and 2B–c). It is energetically advantageous when the hydroxy group fills the neighboring Vo⋅⋅ site to form CH (Schemes 1B–d and 2B–d). In the following *Methyl route*, the barrier for CH_3_ hydrogenation to form CH_4_ is highest (0.75 eV). In comparison, the *Ethene route* via the coupling of CH_2_ was more favorable (*E*_act_ of 0.70 eV, Scheme 2B–e and f), which aligns with the specific formation of ^13^C_2_H_4_ and ^13^C_3_H_6_ from CO within the first 0–4 h of the reaction (Table 1g–i). The isotopic shuffling of ^13^C/^12^C was also plausible between CO adsorbed on the Co^0^ surface and ^12^CO_2_ adsorbed at the Vo⋅⋅ sites from the air (Scheme 1B–a–c).

The advantagenous CO adsorption in the first stage transitions to competitive adsorption of CO (*E*_ads_ of 1.36 eV) and H (*E*_ads_ of 1.32 eV; Scheme 2B–b and b′) in the second step of consecutive photocatalysis. The increased population of H on Co^0^ sites kinetically explains the transition to form C_1–3_ paraffin and C_3_H_6_ after 4 h of the reaction (Figures 2 and S3). In contrast, under CO_2_ and H_2_, H adsorption on Co^0^ is rarely hampered by CO_2_, and CH_2_ is easily transformed to CH_3_ (*E*_act_ of 0.30 eV, Scheme 2A–d) throughout the photocatalytic tests (SI, *3.3. DFT Calculations*).

Selective C_2,3_ olefin formation proceeded during intial 4 h of reaction (Figures 2 and 3) while on later stage, CH_4_ was a major product with minor C_2_H_6_, C_3_H_6_, and C_3_H_8_ (Figure 2). This contrast was understandable if we assume the adsorbed CO/H balance on Co^0^ surface decreased as the time elapsed (Scheme 2B–b and b′). Compared to the two different stages of reaction starting from CO, COH species hopped from Vo⋅⋅ site to Co^0^ surface followed by down‐hill steps mostly to CH_4_ (Scheme 2A–c and d). The consecutive reaction steps on separated sites on ZrO_2_ and Co^0^ starting from CO_2_ proceeded efficiently (Scheme 2A) compared to competitive steps mostly on Co^0^ starting from CO (Scheme 2B and Figure 7).

Photocatalytic CO_2_ reduction has been proposed to proceed via various ways, including the coupling of two CO molecules to form C_2_H_4_ at Vo⋅⋅ sites of TiON,^[16]^ In–(S vacancy)–Bi,^[21]^ and Cu^*δ*+^ sites,^[25]^ the reaction of CO with CHOH to form OC−CHOH and then to C_2_H_4_ at Cu^I^−Cu^II^ sites,^[20]^ and the reaction of CO with COH to form C_2_H_4_ at In_2.77_S_4_ surfaces^[23]^ (Table S1j, n, o, q, and s). However, herein, CO did not directly participate in forming the C−C bond. Instead, the coupling of two species to form C_2_H_4_ at the FeCoS_2_ surface,^[22]^ the coupling of two CHOs to form CH−CHOH and then to C_2_H_4_ at the Fe−N surface,^[24]^ and the coupling of CH_3_ with CH_3_ or C_2_H_5_ to form C_2_H_6_ and C_3_H_8_ at Cu_2_O^[7]^ and Cu surfaces,^[9]^ have been reported. Additionally, the coupling of CH_3_ at TiO_2_ surfaces[^11^, ^12^] and Au surfaces^[13]^ to form C_2_H_6_ has been reported via C−C formation, involving oxygenate and/or HC intermediates (Table S1a, c, e–g, p, and r). In these reports, the combination of reduced active sites with partially oxidized sites resembles the Co^0^ site and the neighboring Vo⋅⋅ site at the ZrO_2_ surface to form C_2,3_‐olefins from CO (Scheme 1B) and C_1–3_‐paraffin from CO_2_ (Scheme 1A).

**Figure 8:**
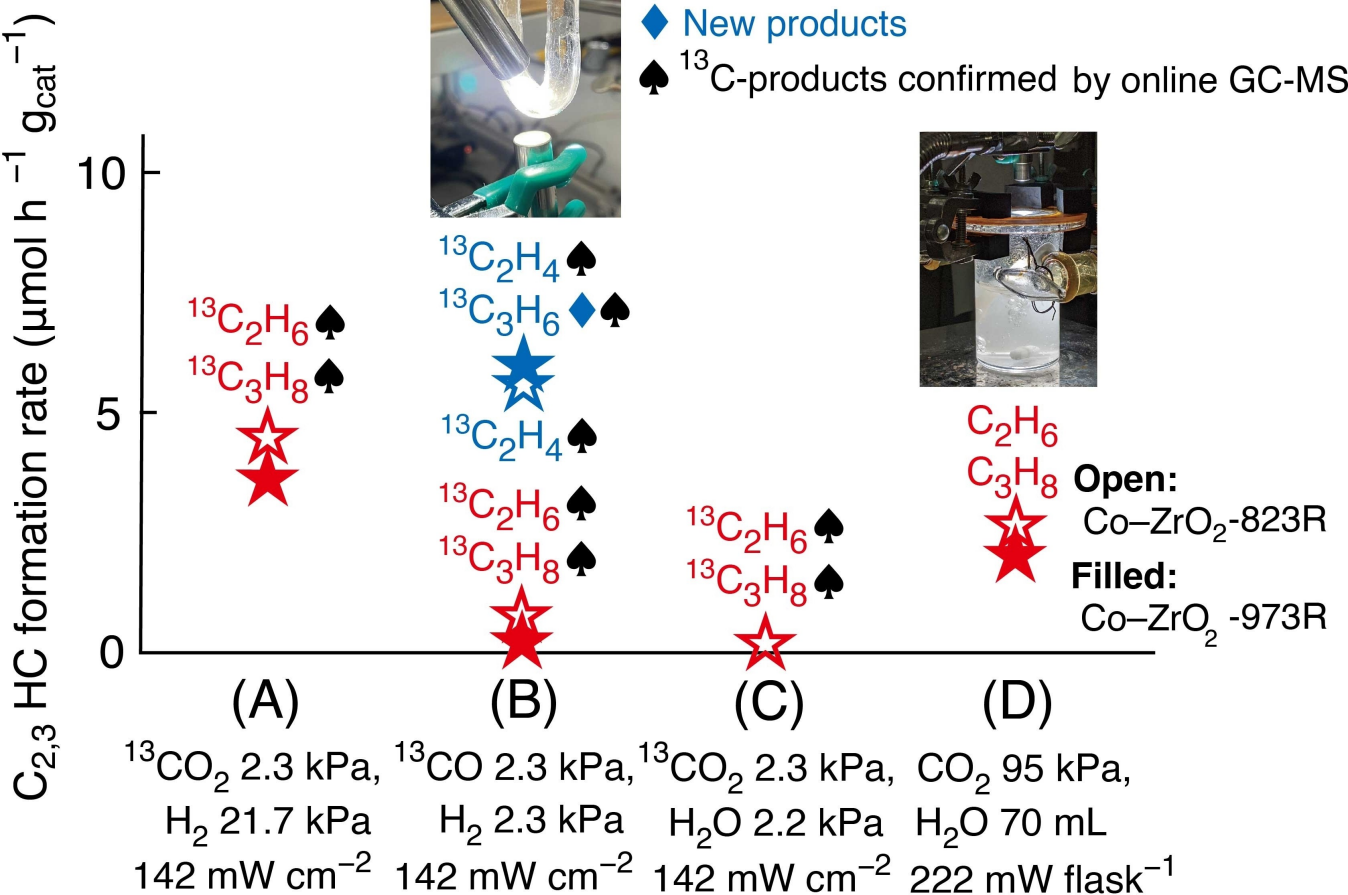
Summary of major findings of C_2,3_ photosynthesis rates using (A, C) ^13^CO_2_, (B) ^13^CO, (D) CO_2_, (A, B) H_2_, (C, D) H_2_O, and Co (7.5 wt %)–ZrO_2_−823R (open symbol) or −973R (filled symbol) photocatalyst irradiated by UV/Visible light at each intensity.

Photocatalytic CO_2_ Reduction Using H_2_O. Compared with the photosynthesis of ^13^C_2_H_6_ and ^13^C_3_H_8_ from ^13^CO_2_ and H_2_ (3.7–4.5 μmol h^−1^ g_cat_^−1^; Table 1c and d and Figure 8A) and the photosynthesis of ^13^C_2_H_4_ and ^13^C_3_H_6_ from ^13^CO and H_2_ (5.5–5.9 μmol h^−1^ g_cat_^−1^; Table 1g and i and Figure 8B), CO_2_ photoreduction was attempted using H_2_O as a one‐step sustainable reaction under reaction conditions similar to those reported in Table S1.[^7^, ^8^, ^9^, ^10^, ^11^, ^12^, ^13^, ^14^, ^15^, ^16^, ^17^, ^18^, ^19^, ^20^, ^21^, ^22^, ^23^, ^24^, ^25^]

The key factors for paraffin synthesis were the metallic Co^0^ surface and the adsorbed H species on the Co^0^. Using the Co−ZrO_2_−823R photocatalyst (Scheme S1A) and swiftly evacuating gas‐phase H_2_, H_2_O (2.2 kPa) served as a reductant for CO_2_ (2.3 kPa) under UV/Visible light irradiation to form ^13^C_2_H_6_ and ^13^C_3_H_8_ at a rate of 0.21 μmol h^−1^ g_cat_^−1^ (Table 1f and Figures 8C and S17). Conversely, O_2_ was not formed above the detection limit because the formed O_2_ reacted with adsorbed H species on the Co surface to regenerate H_2_O at the solid/gas interface.

This challenge was addressed using liquid H_2_O for the photocatalytic CO_2_ conversion to release O_2_ from the surface. C_2_H_6_ and C_3_H_8_ were photogenerated at rates of 2.0–2.7 μmol h^−1^ g_cat_^−1^ at a steady state for 48 h (Figures S18A and B), with up to 60 mol % of C_2,3_ HC formation using H_2_ (Table 1c, k, and l and Figure 8A and D), accompanied by O_2_ formation at the rates higher than 66 μmol h^−1^ g_cat_^−1^ using Co−ZrO_2_−823R and −973 R photocatalysts. The relatively high formation rates to C_2,3_ HCs compared to that to CH_4_ (31–60 mol %; Table 1k and l) were attributed to competitive adsorption of C‐species and H_2_O on Co^0^, rather than the 1.4–1.8 mol % using H_2_ preferably adsorbed on Co^0^ (Table 1c and d). However, sufficiently high light intensity (222 mW cm^−1^) was needed for CO_2_ photoconversion in H_2_O (Tables 1j, l and S8a–e; SI, *3.4. Photocatalytic Conversion of CO_2_ Using H_2_O*).

^13^CO_2_ (2.3 kPa) photoreduction using D_2_O (2.2 kPa), H_2_ (21.7 kPa), and Co−ZrO_2_−823R formed ^13^C‐methane with a D ratio of 9.2 mol %, which agrees with a D ratio in the reactants (9.1 mol %; Figure S19 and Table S7b; SI, *3.5. Photocatalytic Conversion of* ^*13*^ *CO_2_ Using D_2_O*). This suggests that D_2_O and H_2_ reached equilibrium more rapidly and were shuffled over Co^0^ more efficiently than the progressive hydrogenation steps to C_2,3_ paraffin common under either H_2_ or H_2_O (Scheme 1A–a–d). Such a photocatalytic pathway from CO_2_ to C_2,3_ paraffin using either H_2_ or H_2_O[^7^, ^8^, ^9^, ^10^, ^11^, ^12^, ^13^, ^14^, ^15^] and the photoformation from CO to C_3_H_6_ have been rarely reported.

This study paves the way to precisely explore further active photocatalysts to selectively produce C_2,3_‐HCs utilizing unsaturated/lower‐dimensional semiconductors to regenerate the Vo⋅⋅ sites with an *E*_act_ of <2.6 eV (Scheme 2B). This can be achieved in combination with metal nanoparticles/single atoms using either H_2_ or H_2_O, surpassing the capabilities of recent photocatalysts (Table S1).[^7^, ^8^, ^9^, ^10^, ^11^, ^12^, ^13^, ^14^, ^15^, ^16^, ^17^, ^18^, ^19^, ^20^, ^21^, ^22^, ^23^, ^24^, ^25^]

## Conclusions

The Co (7.5 wt %)–ZrO_2_−823R photocatalyst formed C_1–3_ paraffin at a total formation rate of 330±20 μmol h^−1^ g_cat_^−1^ using CO_2_ and H_2_. In contrast, Co (7.5 wt %)–ZrO_2_−973R formed C_2_H_4_ and C_3_H_6_ at a total formation rate of 6.0±0.6 μmol h^−1^ g_cat_^−1^ with an olefin selectivity of 70 mol % using CO and H_2_. CO_2_ was adsorbed on the Vo⋅⋅ sites at the ZrO_2_ surface, and the intermediate COH species hopped onto the Co^0^ surface, where it was progressively hydrogenated to form CH_3_ and/or CH_2_ species, which subsequently coupled to form C_1–3_ paraffin. Conversely, CO adsorbed on Co^0^ was hydrogenated to form HOCH and was most effectively dissociated to CH at the interface with the ZrO_2_ surface comprising the Vo⋅⋅ site. Preferential CO adsorption and favorable CH_2_ coupling until 4 h were followed by the competitive adsorption of CO and H on Co^0^ sites, resulting in consecutive CH_4_ and C_3_H_6_ formation. Predominant photocatalytic formation of C_2_H_4_ and C_3_H_6_ (61–87 mol %) was achieved from CO after repeated tests for 4–10 h, followed by evacuation. The dual mechanism involved electron donation from Vo⋅⋅ to OCOH/HOCH species using CO_2_/CO, followed by consecutive hydrogenation steps on the Co^0^ surface at 362/350 K, facilitated by UV/Visible light energy, at a rate of 200/71 000 times higher than that observed via a thermal reaction at 362/350 K, respectively, without light. CO_2_ photoreduction to C_2_H_6_ and C_3_H_8_ was possible in H_2_O (l) to release O_2_ from the surface via a very similar pathway involving CH_3/2_ activation on Co^0^.

## Conflict of Interests

The authors declare no conflict of interest.

1

## Supporting information

As a service to our authors and readers, this journal provides supporting information supplied by the authors. Such materials are peer reviewed and may be re‐organized for online delivery, but are not copy‐edited or typeset. Technical support issues arising from supporting information (other than missing files) should be addressed to the authors.

Supporting Information

## Data Availability

The data that support the findings of this study are available from the corresponding author upon reasonable request.
