# Supplementary material for: Exchange of CO2 with CO as Reactant Switches Selectivity in Photoreduction on Co−ZrO2 from C1–3 Paraffin to Small Olefins
Source: Angew Chem Int Ed Engl. 2024 Nov 2;63(51):e202412090. doi: 10.1002/anie.202412090 (PMC11627130; doi:10.1002/anie.202412090)
Supplement: Supplementary file 1 — Supporting Information [file ANIE-63-e202412090-s001.pdf]

Angewandte Chemie
Eine Zeitschrift der Gesellschaft Deutscher Chemiker

Supporting Information

Exchange of CO_2 with CO as Reactant Switches Selectivity in Photoreduction on Co–ZrO_2 from $C_{1–3}$ Paraffin to Small Olefins

*T. Loumissi, R. Ishii, K. Hara, T. Oyumi, I. Abe, C. Li, H. Zhang, R. Hirayama, K. Niki, T. Itoi, Y. Izumi**

Exchange of CO_2 with CO as Reactant Switches Selectivity in Photoreduction on Co–ZrO_2 from $C_{1–3}$ Paraffin to Small Olefins

Tarik Loumissi,[a],# Rento Ishii,[a],# Keisuke Hara,[a],# Tomoki Oyumi,[a],# Ikki Abe,[a] Chongxu Li,[a] Hongwei Zhang,[a] Rumiko Hirayama,[a] Kaori Niki,[a] Takaomi Itoi,[b] and Yasuo Izumi*[a]

[a] T. Loumissi, R. Ishii, Dr. K. Hara, T. Oyumi, I. Abe, C. Li, Professor Dr. H. Zhang, R. Hirayama, Professor Dr. K. Niki, and Professor Dr. Y. Izumi
Department of Chemistry, Graduate School of Science
Chiba University
Yayoi 1-33, Inage-ku, Chiba 263-8522, Japan
E-mail: yizumi@faculty.chiba-u.jp

[b] Professor Dr. T. Itoi
Department of Mechanical Engineering, Graduate School of Engineering
Chiba University
Yayoi 1-33, Inage-ku, Chiba 263-8522, Japan

T.L., R.I., and T.O. shared experiments and K.H. contributed for calculations equally to this paper all in the graduate course.

1. INTRODUCTION

1.1. Background of This Study.

Table S1. Reported Photocatalytic C_2 and C_3 HC Generation from CO_2.

Entry	Catalyst	Light	Reactants	Solvent/reactant	Reaction time	Product	Formation rate and selectivity	Year	Ref
a	Co–Cu/TiO_2	300-W Xe	CO_2 (1.0 MPa)	Water (1 mL)	3 h	C_2H_6	270 μmol h^{-1} g_{cat}^{-1}, 45 mol%	2019	S1
						C_3H_8	10 μmol h^{-1} g_{cat}^{-1}, 1.7 mol%		
b	Single Au/red P	300-W Xe (300 mW cm^{-2})	$KHCO_3$ + HCl → CO_2 + H_2O + KCl	–	–	C_2H_6	1.3 μmol h^{-1} g_{cat}^{-1}, 96 mol%	2022	S2
c	CdS/Cu–titanate	450-W Xe, >420 nm	CO_2 (101 kPa)	Water (25 mL)	–		0.71 μmol h^{-1} g_{cat}^{-1}, 31 mol%	2015	S3
d	Nafion–Pd–TiO_2	Sun, 36° north		aq. Na_2CO_3, 1.5 g_{cat} L^{-1}	6 h		1.1 μmol h^{-1}, 9.0 mol%	2012	S4
e	Pt–graphene/TiO_{2-x}	100 W, AM1.5 filter (100 mW cm^{-2})	CO_2 flow (101 kPa)	CO_2 flows through H_2O	7 h		11 μmol h^{-1}, 23 mol%	2018	S5
f	TiO_2–graphene	300-W Xe	CO_2 (101 kPa)	Water (0.4 mL)	4 h		17 μmol h^{-1} g_{cat}^{-1}, 68 mol%	2013	S6
g	Au@$Bi_{12}O_{17}Br_2$	300-W Xe	CO_2 (80 kPa)	Water (1 mL)	20 h		29 μmol h^{-1} g_{cat}^{-1}, 59 mol%	2022	S7
h	$CuPt_2/TiO_2$ nanotube	AM1.5 (100 mW cm^{-2})	CO_2 flow (650 kPa)	Water (15 μL)	–	C_2H_6	20 μmol h^{-1} g_{cat}^{-1}; 14 mol%	2012	S8
						C_2H_4	10 μmol h^{-1} g_{cat}^{-1}; 7.1 mol%		
i	Au–Pd/TiO_2{1 0 1}	300-W Xe (853 mW cm^{-2})	CO_2 (0.26 MPa)	Water (0.1 mL)	6 h	C_2H_6	0.79 μmol h^{-1} g_{cat}^{-1}, 3.2mol%	2019	S9
						C_2H_4	0.73 μmol h^{-1} g_{cat}^{-1}; 2.9 mol%		
j	$TiON_{1-(O\ vacancy)}$	Xe, > 420 nm (40 mW cm^{-2})	CO_2 (101 kPa)	The catalyst was immersed in water and dried in vacuum	4 h		1.9 μmol h^{-1} g_{cat}^{-1}, 2 mol%	2023	S10
k	C/Cu_2O nanorod	350-W Xe >420 nm		0.1 M $KHCO_3$	12 h		0.016 μmol h^{-1}, 56 mol%	2016	S11
l	Cu/TiO_2	Xe	CO_2 (2.7 MPa)	–	–		0.023 μmol h^{-1} g_{cat}^{-1}, 49 mol%	1994	S12
m	Ag–C nanotube @TiO_2	Visible light	CO_2 (101 kPa)	CO_2 flows through H_2O	7.5 h		0.048 μmol h^{-1} g_{cat}^{-1}, 5 mol%	2015	S13
n	CuO/$CuGaS_2$	450-W Xe UV cut filter		aq. NaOH (pH 12)	16 h		20 μmol h^{-1} g_{cat}^{-1}, 75 mol%	2023	S14
o	Bi_2S_3@In_2S_3	300-W Xe (1-W cm^{-2})		Water (1 mL)	5 h		12 μmol h^{-1} g_{cat}^{-1}, 86 mol%		S15
p	$FeCoS_2$	300-W Xe AM 1.5-G filter		The catalyst immersed in water, dried at 333 K	10 h		20 μmol h^{-1} g_{cat}^{-1}, 83 mol%		S16
q	$In_{2.77}S_4$/porous polymer	450-W Xe (110 mW cm^{-2})		Water (20 mL)	10 h		68 μmol h^{-1} g_{cat}^{-1}, 14 mol%		S17
r	N,S/Fe–MOF	300-W Xe (200 mW cm^{-2}), >420 nm		Water (2 mL)	4 h		18 μmol h^{-1} g_{cat}^{-1}, 11 mol%		S18
s	$Cu^{\delta+}/CeO_2$–TiO_2	Xe, 200 mW cm^{-2}		Water (30 mL)	5 h		4.5 μmol h^{-1} g_{cat}^{-1}, 47 mol%	2022	S19

1.2. Major Framework of This Study.

Table S2. Summary of Kinetic Data and the Monitoring on Photoconversion of CO_2 and/or CO Using the ZrO_2 and Co–ZrO_2 Photocatalysts in This Study.

Entry	Catalyst	Pretreat *T* (K) under H_2	Reactants: C oxide	Reactants: Reductant-1	Reactants: Reductant-2	Light irradiated /heat applied (*T*) *1	Table	Figure
a	ZrO_2	823	$^{13}CO_2$ (2.3 kPa)	H_2 (21.7 kPa)	none	full light	S3	
b				H_2 (2.3 kPa)				
c		none	CO_2 (95 kPa)	H_2O (70 mL)			S8	
d	Co (2.5 wt %)–ZrO_2	823	$^{13}CO_2$ (2.3 kPa)	H_2 (21.7 kPa)			S3	
e	Co (5.0 wt %)–ZrO_2							
f	Co (7.5 wt %)–ZrO_2	none					2, S4	
g		723						S2A
h		823	$^{13}CO_2$ (0.68 kPa)	none			S5	4
i			$^{13}CO_2$ (2.3 kPa)	H_2 (21.7 kPa)			2, S3 S4, S7	1, 8, S2B, S8c (the best for $C_{1–3}$ paraffins)
j			N_2 (2.3 kPa)				S3	
k			$^{13}CO_2$ (2.3 kPa)			$\lambda > 320$ nm		
l						$\lambda > 520$ nm		
m						363 K	S6A	Scheme 1D
n						393 K		
o						423 K		
p					D_2O (2.2 kPa)	full light	S7	S19; S21 (EXAFS)
q		973			none		2, S4	8, S2C; 6, S11, S12, S13 (XAFS) (2nd best for $C_{1–3}$ paraffins)
r				H_2 (2.3 kPa)				
s		823		H_2O (2.2 kPa)				8, S17
t			CO_2 (95 kPa)	H_2O (70 mL)			2, S8	8, S18 ($C_{1–3}$ paraffin formation using H_2O)
u		973						
v	Co (10 wt %)–ZrO_2	823	$^{13}CO_2$ (2.3 kPa)	H_2 (21.7 kPa)			S3	
w	Co (7.5 wt %)–ZrO_2	823	^{13}CO (2.3 kPa)	H_2 (2.3 kPa)			2, S4	8, S3, S4
x				H_2O (2.3 kPa)				S20
y		973		H_2 (21.7 kPa)			2, S4	
z				H_2 (2.3 kPa)				2, 3, 8, S5, Scheme 1D; S14, S15 (EXAFS); 7, S16 (FTIR) (the best for $C_{2,3}$ olefins)
α						363 K	S6B	Scheme 1D
β						393 K		
γ						423 K		

*1 The photocatalyst was irradiated by light without any external heat applied or it was applied by external heat under dark.

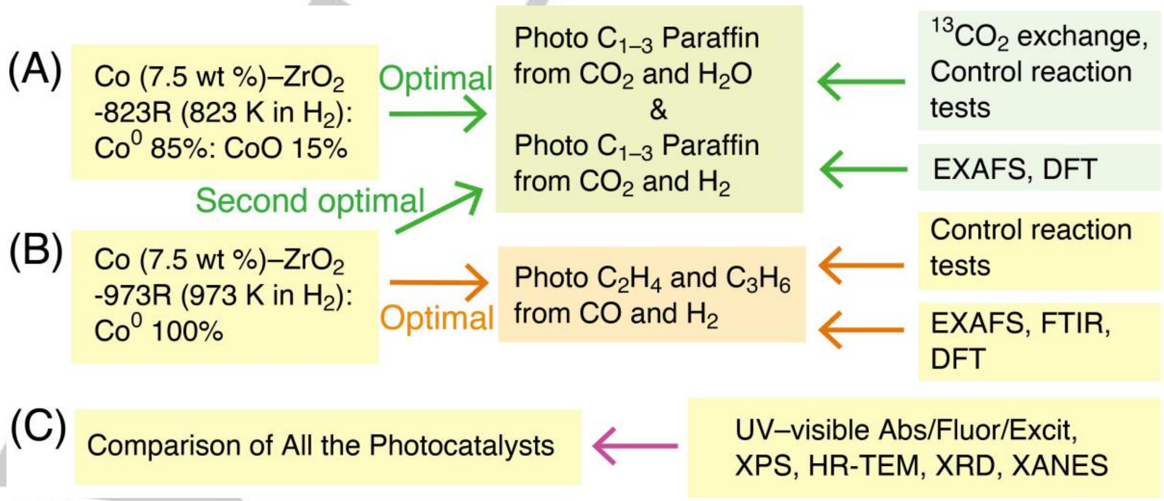

Scheme S1. Correlation among Photocatalyst Composition, Photocatalytic Products, and Methods Utilized in This Study (Table S2i, q, t, u, and z).

Table S2 summarizes kinetic data and the characterization results of this study. Among the tested photocatalysts, Co (7.5 wt %)–ZrO_2-823R (where 973 denotes the pretreatment temperature (K) of the photocatalysts with H_2 and R represents reduced) photocatalyst exhibited the highest activity for the formation of $C_{1–3}$ paraffin using CO_2, H_2 and/or H_2O, and UV–visible light irradiation (Table S2i and t and Scheme S1A). We investigated the photocatalytic reaction pathway by monitoring isotope-labeled kinetic monitoring using $^{13}CO_2$ and comparing them with thermal reactions. To simplify the discussion for the pathway over Co species, the second active Co (7.5 wt %)–ZrO_2-973R photocatalyst comprising only Co^0 sites, was well characterized via extended X-ray absorption fine structure (EXAFS) and density functional theory (DFT) calculations (Table S2q and Scheme S1B).

The Co (7.5 wt %)–ZrO_2-973R photocatalyst exhibited the highest activity for the formation of C_2H_4 and C_3H_6 formation using CO, H_2, and UV–visible light irradiation. We investigated the consecutive photocatalytic reaction pathway using ^{13}CO-labeled species and/or repeated kinetic tests, alongside comparison with thermal reaction, EXAFS, Fourier-transform infrared (FTIR) spectroscopy, and DFT (Table S2z and Scheme S1B).

All tested photocatalysts were compared using UV–visible absorption/fluorescence/emission, X-ray photoelectron spectroscopy (XPS), high-resolution transmission electron microscopy (HR-TEM), X-ray diffraction (XRD), and X-ray absorption near-edge structure (XANES) analyses (Scheme S1C) to identify the key control factors influencing CO_2/CO photoreduction using Co–ZrO_2 photocatalysts.

2. EXPERIMENTAL SECTION

ZrO_2 (0.500 g, JRC-ZRO-3, Catalysis Society of Japan, predominantly monoclinic phase, specific surface area = 99.4 m^2 g^{-1}) was dispersed in deionized water (< 0.055 μS cm^{-1}) supplied by a model RFU424TA (Advantec, Japan), and 0.0633–0.274 g of Co nitrate hexahydrate (purity > 99.5%, Wako Pure Chemical, Japan) was added. The suspension was ultrasonicated for 20 min (430 W, 38 kHz) and stirred at a rate of 900 rpm. $NaBH_4$ (0.0658–0.285 g, $NaBH_4$: Co molar ratio = 8: 1) dissolved in an aqueous solution (20 mL) was added within 1 min and stirred for 10 min at a rate of 900 rpm. The resulting precipitate was filtered using a membrane filter (pore size = 0.1 μm; Omnipore JVWP04700, Merck–Millipore, Darmstadt) and washed with deionized water (50 mL, five times). The resulting green powder was dried at 373 K for 24 h and then heated in H_2 (21.7 kPa) at 723 K for 10 min or at 823 or 973 K for 1 h. The prepared samples comprising x wt % (x = 2.5–10) of Co reduced at T K under H_2, were denoted as Co (x wt %)–ZrO_2-TR.

The CO_2 photoreduction tests at the gas/solid interface were conducted using 20 mg of the photocatalysts. $^{13}CO_2$ (2.3 kPa, purity > 99.9%; 99.0% ^{13}C, 0.1% ^{17}O, 0.7% ^{18}O, Cambridge Isotope Laboratories, Inc., Tewksbury, MA, USA) or ^{13}CO (2.3 kPa, purity 99.5%; 99% ^{13}C, < 2% ^{18}O, Cambridge Isotope Laboratories, Inc.) and H_2 (2.3–21.7 kPa; purity > 99.99%) and/or H_2O (2.2 kPa) were used. The U-shaped quartz reactor containing the catalyst (Chart S1A) was irradiated with UV–visible light emitted from a 500 W Xe arc lamp (Model SX-UID502XAM, Ushio, Japan) through a Y-shaped quartz light guide (Model 1.2S15-1000F-1Q7-SP-RX; a 40 cm-long fiber and 80 cm-long branches; Optel, Tokyo, Japan).[S20] The distance between the fiber light exit and the photocatalyst was 2 cm (Chart S1A). The light intensity at the center of the sample was 142 mW cm^{-2} (Table 1a–i).

Light transmission was monitored using a photosensor (Model PCM-01, Prede, Tokyo, Japan) and a counter (Model KADEC-UP, North One, Sapporo, Japan). The photosensor was placed on the opposite side of the fiber light exit, with a distance of 2 cm between the photosensor (light entrance) and the photocatalyst (Chart S1B and C). Approximately 60.4% ± 0.1% of light (300 nm < wavelength λ < 2800 nm) from one of the fibers (71 mW cm^{-2}) transmitted through both sides of the quartz reactor, resulting in 77.7% ± 0.1% transmission on one side of the reactor. The transmitted light was through ZrO_2, Co (7.5 wt %)–ZrO_2-fresh (Chart S1B), Co (7.5 wt %)–ZrO_2-723R, and Co (7.5 wt %)–ZrO_2-973R (Chart S1C) located in the reactor (each 20 mg) was 3.3% ± 0.2%, 4.1% ± 0.3%, 4.1% ± 0.2%, and 1.7% ± 0.1%, respectively. Thus, ZrO_2 powder and Co (7.5 wt %)–ZrO_2-fresh, -723R, and -973R samples reflected/scattered/absorbed 94.5 ± 0.3%, 93.2% ± 0.5%, 93.2% ± 0.3%, and 97.2% ± 0.2% of light, respectively. Based on the UV–visible absorption spectra (Figure S6A) and the color of samples, while ZrO_2 powder mostly reflected/scattered the light, Co_3O_4, CoO, and Co^0 nanoparticles in dark green, dark brown, and black Co (7.5 wt %)–ZrO_2-fresh, -723R, and -973R samples, respectively, mostly absorbed the light.

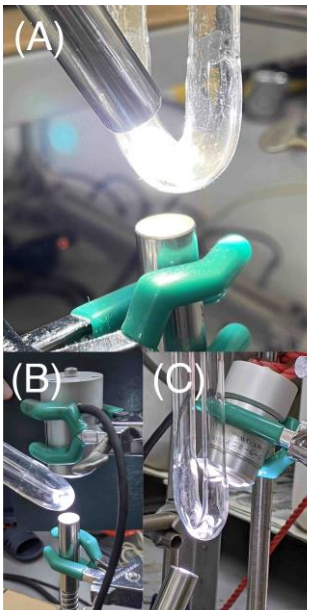

Chart S1. (A) Photocatalyst in Quartz Reactor Irradiated with UV–Visible Light from 500 W Xe Arc Lamp via Two Branches of Quartz Light Guide. Light intensity 142 mW cm^{-2}. (B, C) Transmitted Light Intensity Detected by a Photosensor through (B) ZrO_2 and Co (7.5 wt %)–ZrO_2-fresh and (C) Co (7.5 wt %)–ZrO_2-723R and -973R in Quartz Reactor Irradiated via a Branch of Quartz Light Guide.

The CO_2 photoconversion tests were also conducted using CO_2 (95 kPa) and H_2O (70 mL) with $NaHCO_3$ (0.202 g, purity > 99.5%; Wako Pure Chemical, Japan) dissolved in it. Photocatalyst samples (2.2–37 mg), pretreated under H_2, were sealed using fire and transferred to this reactor using the Schlenk technique under the Ar atmosphere. CO_2 (95 kPa) was circulated within a closed Pyrex glass system connected to a Pyrex flask equipped with a quartz window (Chart S2) containing the photocatalyst (2.2–37 mg). The reactor was then

WILEY

irradiated with UV–visible light using either a 500 W Xe arc lamp (Model OPM2-502, Ushio, Japan) through a light guide with a diameter of 5 cm or a 300 W Xe arc lamp (Model MAX-350, Asahi Spectra Co., Tokyo, Japan) through a quartz light guide. The distance between the former light exit and the quartz window was 4 cm while that between the latter fiber light exit and the photocatalyst was 2 cm (Chart S2). The light intensity at the center of the sample was measured at 90.2 mW per flask (Table 1j) and 222 mW per flask, respectively (Table 1k and l). The suspension was stirred at 1 000 rpm, and the reaction gas was bubbled using a gas circulation pump connected to the reactor during the photocatalytic reaction tests (Chart S2).

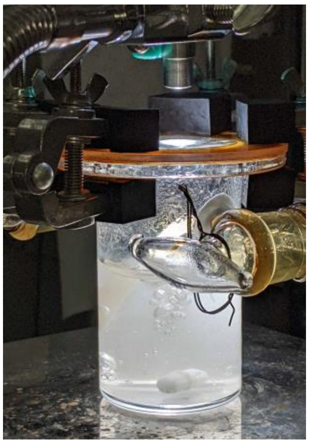

Chart S2. Photocatalytic Pyrex Flask Reactors Containing H_2O (70 mL) Equipped with Quartz Window on the Top Irradiated with UV–Visible Light from a 300 W Xe Arc Lamp through a Quartz Light Guide. Light intensity 222 mW per flask.

For online gas chromatography–mass spectrometry (GC–MS; Model JMS-Q1050GC, JEOL, Japan) for photocatalytic reaction tests, a packed column of 13X-S molecular sieves (length = 3 m, internal diameter = 3 mm; GL Sciences, Inc., Japan) was employed. Additionally, a packed column of polyethylene glycol-6000/Flusin P (length = 3 m, internal diameter = 3 mm; GL Sciences, Inc.) was employed for HCO_2H, CH_3OH, C_2H_5OH, $(CH_2OH)_2$, and iso-C_3H_7OH and $^{13}CO_2$ photo-uptake/exchange reaction tests both using He (purity > 99.99995%) as the carrier gas. The ionization energy of separated gas was 20 eV. Detection of $^{13}CH_4$ and $^{12}CH_4$ relied on the mass number (*m*/*z*) of 17 and 16, respectively, considering the fragment ratio of CH_3^+:CH_4^+ (0.708:1).[S20] Similarly, ^{13}CO, $^{13}C_2H_6$, $^{13}C_2H_4$, $^{13}C_3H_8$, $^{13}C_3H_6$, HCO_2H, CH_3OH, C_2H_5OH, $(CH_2OH)_2$, and iso-C_3H_7OH were evaluated based on the fragment peak of *m*/*z* = 29 ($^{13}CO^+$), 30 ($^{13}C_2H_4^+$), 30 ($^{13}C_2H_4^+$), 31 ($^{13}C_2H_5^+$), 44 ($^{13}C_3H_5^+$), 46 (HCO_2H^+), 31 (CH_3O^+), 31 (CH_3O^+), 62 ($(CH_2OH)_2^+$), and 45 ($C_2H_5O^+$), respectively, in each mass chromatogram. Under the analysis conditions in this study using the 13X-S molecular sieves packed column, the mixture of ^{13}CO and $^{13}CO_2$ was well separated[S21] and no ^{13}CO (*m*/*z* = 29) was detected throughout the mass chromatogram when pure $^{13}CO_2$ was analyzed. Furthermore, all the reactants and products were uniquely quantified based on the predominant peak (GC retention time) in each mass chromatogram.

For photocatalytic tests using H_2O (70 mL), the products were analyzed using an online GC-thermal conductivity detector (Model GC-8AT, Shimadzu, Kyoto, Japan) equipped with a packed column of 13X-S molecular sieves (length 3 m, internal diameter = 3 mm) using He (purity > 99.99995%) as the carrier gas.

The photocatalytic products in 70 mL of H_2O (liquid) were also analyzed using a 500 MHz ^1H nuclear magnetic resonance (NMR) spectrometer (Model JNM-ECA500, JEOL, Japan) at the Center for Analytical Instrumentation of Chiba University, Japan, employing the solvent suppression method. Before the excitation with a rectangular 45° pulse with an irradiated rotational magnetic field intensity B_1 (16.7 kHz), the pre-saturation at 4.67 ppm (4 s, B_1 = 25 Hz) was applied to attenuate the peak from H_2O. A solution of 700 μL of photocatalytic reaction in ^1H_2O solution was mixed with 35 μL of D_2O (D 99.8%, Wako Pure Chemical) solution containing 10 mM of dimethyl sulfoxide (> 99.0%, Wako Pure Chemical) as the internal standard.

In-profile kinetic data were collected as a function of light excitation wavelength by inserting sharp-cut filters (2.5 mm thick) at the light fiber exits. The UV32 (or UV32N) and Y52 sharp-cut filters (Hoya, Japan) were used to pass light with λ > 320 nm and > 520 mm, respectively.

UV–visible spectra were recorded on a double-beam model V-650 spectrometer using D_2 and halogen lamps below and above 340 nm equipped with a photomultiplier tube and an integrated ISV-469 sphere (JASCO, Tokyo, Japan) for diffuse-reflectance detection within the range from 200 to 800 nm. Samples treated under H_2 were transferred to an airtight cell without contact with air, and a formed polytetrafluoroethylene plate was used as a reference.

XP spectra were measured using KRATOS ULTRA2 (Shimadzu, Kyoto, Japan) equipped with an Al Kα light source for Co (7.5 wt %)–ZrO_2-723R, Co (7.5 wt %)–ZrO_2-823R, and Co (7.5 wt %)–ZrO_2-823R under conditions of 2.3 kPa of CO_2 and 21.7 kPa of H_2. The energy was calibrated to 285.0 eV for C–H at C 1s.

The absorption–fluorescence spectra were recorded on an FP-8600 spectrometer (JASCO; Chiba Iodine Resource Innovation Center, Chiba, Japan) equipped with a 150 W Xe arc lamp (UXL-159, Ushio) and a photomultiplier tube for excitation at 200–450 nm within a fluorescence range of 300–800 nm. The incident excitation light from the Xe lamp was monitored using a Si photodiode, and the monitored fluorescence light emitted from the sample was normalized based on the incident light intensity at each wavelength. The photocatalyst powder (2.0 mg) was mixed with deionized water (< 0.055 μS cm^{-1}; 3.0 mL) and ultrasonicated (430 W, 38 kHz) for 30 min. All spectra were recorded for the suspensions in a quartz cell at 295 K.

HR-TEM was performed using a JEM-2100F (JEOL) equipped with a field-emission gun (acceleration voltage of 200 kV) at the Center for Analytical Instrumentation. The samples were mounted on a Cu mesh (250 meshes per inch) coated with carbon and a copolymer film of poly(vinyl alcohol) and formaldehyde (Formvar, Monsanto, St. Louis, MO, USA).

XRD patterns were observed using a D8 ADVANCE diffractometer (Bruker, Billerica, MA, USA) at the Center for Analytical Instrumentation with a Bragg angle (θ_B) of $2\theta_B$ = 20°–60°, employing a scan step of 0.02° and scan rate of 0.5 s per step. The measurements were performed at 40 kV and 40 mA using Cu Kα emission (λ = 0.15419 nm) with a Ni filter.

Cobalt K-edge XAFS spectra were measured in the transmission mode at the Photon Factory, High Energy Accelerator Research Organization (Tsukuba, Japan) on the

9C beamline using a Si (1 1 1) monochromator, a Pt-coated mirror, and a piezo transducer.[S20,S22] The Co–ZrO_2 samples were treated in a quartz U-tube and transferred to a Pyrex cell filled with reaction gases, which was equipped with a polyethylene terephthalate film (Toyobo, Japan, G2, 50 μm thick) on both sides for both UV–visible light and X-ray transmission. A 500 W Xe arc lamp (Model SX-UID502XAM) was used as the light source, with the distance between the fiber light exit of the Y-shaped quartz light guide and the photocatalyst set at 2 cm.

The obtained Co K-edge XAFS data were analyzed using the XDAP software package version 3.2.9.[S23] The pre-edge background was approximated with a modified Victoreen function, $C_2/E^2 + C_1/E + C_0$, where E is the photon energy and C_0, C_1, and C_2 are constants. Multiple-shell curve-fit analyses were performed with the data obtained on the Fourier-filtered angular wave number k^3-weighted EXAFS using the empirical amplitude extracted from the EXAFS data for the Co metal foil (10 μm thick) and CoO powder. The interatomic distances and coordination number values for the Co–Co and Co–O interatomic pairs were set to 0.2502 nm and 12[S24] and 0.2131 nm and 6,[S25] respectively.

The temperature dependence of the Debye–Waller factor value was calculated for the bulk and surface sites of Co metal (Figure S1) using the correlated Debye model with the ab initio multiple-scattering calculation code, FEFF version 8.4.[S26] The bulk and surface Co site temperatures were determined (see the main text, *Photothermal Monitoring and the Control Thermal* $^{13}CO_2/^{13}CO$ *Reduction* section).

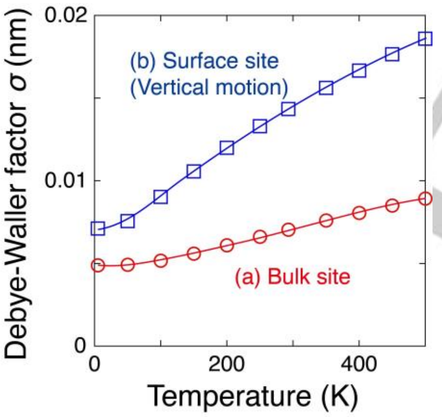

Figure S1. Correlation between the Debye–Waller factor and temperature for (a) bulk sites (circle, ○) and (b) surface sites (vertical motion; square, □) in/on the Co metal, calculated using the correlated Debye model with a FEFF version 8.4 code.[S26]

In situ FTIR spectroscopy measurements were performed at 293 K, ranging from 4000 to 650 cm^{-1}, using a model FT/IR-4200 instrument (JASCO, Tokyo, Japan). The Co–ZrO_2 photocatalyst disk (71 mg) was heated under H_2 in a quartz cell and transferred to an FTIR cell using a glove box (UN-650LCIY, Unico, Japan). The sample disk was irradiated with UV–visible light from a 500 W Xe arc lamp (Model SX-UID502XAM) through a quartz fiber light guide. The distance between the fiber light exit and the sample disk was 5 cm. The energy resolution of the spectrometer was set to 1 cm^{-1}, and data accumulation was performed for 512 scans (~2 s per scan).

Spin-polarized periodic DFT calculations were conducted using the Vienna Ab initio Simulation Package code version 6.2.1,[S27] computed on a VT64 Server XS2-2TI comprising four units of Intel Xeon Platinum 9242 Processors (2.3 GHz, 48 cores; Visual Technology, Tokyo, Japan) and partially utilizing the facilities of the Supercomputer Center at the Institute for Solid State Physics, the University of Tokyo, Japan. The projector-augmented-wave method was employed at the DFT-D3 level[S28] to incorporate the van der Waals interactions. The generalized gradient approximation-revised Perdew–Burke–Ernzerhof exchange–correlation functional[S29] was employed with a cutoff set at 500 eV, and Hubbard U parameter of 4.0 eV for Zr was added.[S30,S31] Transition states were pursued using the climbing image-nudged elastic band method.[S32]

Hemispherical Co_{19} clusters exposing the (0 1 0) [= (0 1 $\bar{1}$ 0)] face based on the hexagonal closest-packed (hcp) structure of Co metal were used combined with monoclinic ZrO_2 (1 1 1) face comprising (2 × 2 × 2) element unit cells as a slab model with a vacuum spacing of 2.0 nm between the slabs in the direction of the [1 1 1] axis of ZrO_2.[S33]

3. RESULTS AND DISCUSSION

3.1. Kinetic Results. The uncertainty in the amount of product observed in the photocatalytic time course (Figures 1–3 and S3) stemmed from several factors, and all the following factors were taken into accounts to evaluate the data errors.

(a) The error of online GC–MS evaluation connected to a circulating system comprising a U-shaped quartz photoreactor (Chart S1A): 0.046%, 0.031%, 0.43%, 0.070%, 0.070%, 0.16%, and 0.16% for ^{13}CO, $^{13}CH_4$, $^{12}CH_4$, $^{13}C_2H_6$, $^{13}C_2H_4$, $^{13}C_3H_8$, and $^{13}C_3H_6$ using a packed column of 13X-S molecular sieves, based on calibration line.
(b) The error assuming first-order reaction kinetics due to reactant loss during sampling (1.8 volume-% each): 0.0051% and 0.0794% using ($^{13}CO_2$ and H_2) and (^{13}CO and H_2), based on the comparison between five-point sampling and one-point sampling for 5 h.
(c) The error among three photocatalytic kinetic tests: 0.57–85%, 8.2–20%, 13–29%, 13–58%, and 18–101% for ^{13}CO, $^{13}CH_4$, $^{12}CH_4$, $^{13}C_2H_6$, and $^{13}C_3H_8$ formations using $^{13}CO_2$ and H_2 (Figure 1) and 3.3–9.0%, 0.96–60%, 15–46%, 10–90%, 14–87%, 21–143%, and 5.9–174% for ^{13}CO, $^{13}CH_4$, $^{12}CH_4$, $^{13}C_2H_4$, $^{13}C_2H_6$, $^{13}C_3H_6$, and $^{13}C_3H_8$ formations using ^{13}CO and H_2 (Figures 2 and S3) owing to the differences in nonadjustable reaction conditions, e.g., the storage duration of $Co(NO_3)_3 \cdot 6H_2O$ reagent under Ar at 273 K, the duration since the synthesis of photocatalyst synthesized was kept under Ar, and more critically, the nonlinear correlation between product formation rates and excitation light intensity.

More than 87% of the formation rates were consistent in repeated photocatalytic tests under $^{13}CO_2$ and H_2 [(i) a set of pretreatment and the 48-hour kinetic test was repeated three times, (ii) a 96-h kinetic test] except for a test conducted after a 48-hour kinetic test and a subsequent 1-hour evacuation. In the latter reaction, the formation of $^{13}CH_4$ and $^{13}C_2H_6$ formation decelerated after 2 h of reaction owing to the presence of HC intermediate remaining on the Co surface, while H was removed after evacuation, leading to an imbalance in the surface population of CO/COH/HC/H species necessary for the HC formation (see FTIR spectroscopy section in main text).

Next, in-profile CO_2 reduction tests were performed. By increasing the minimum wavelength for irradiation to 320 nm and then 520 nm, the rate of $^{13}CH_4$ formation gradually

decreased to 43% and 22% of that observed under full light (Table S3e, g, and h). The ratio of $^{13}CH_4$, $^{12}CH_4$, $^{13}C_2H_6$, to $^{13}C_3H_8$, remained almost constant at 100: 4.6–7.7: 1.3–2.0: 0.14–2.6, regardless of the wavelength region of the irradiated light. This suggests that instead of band-gap excitation (5.0 eV; Figure S6A), the vacancy/impurity level of ZrO_2 (Figure S6B and C) played an auxiliary role in charge separation for reducing CO and subsequent reduction mechanisms over Co sites to form HCs.

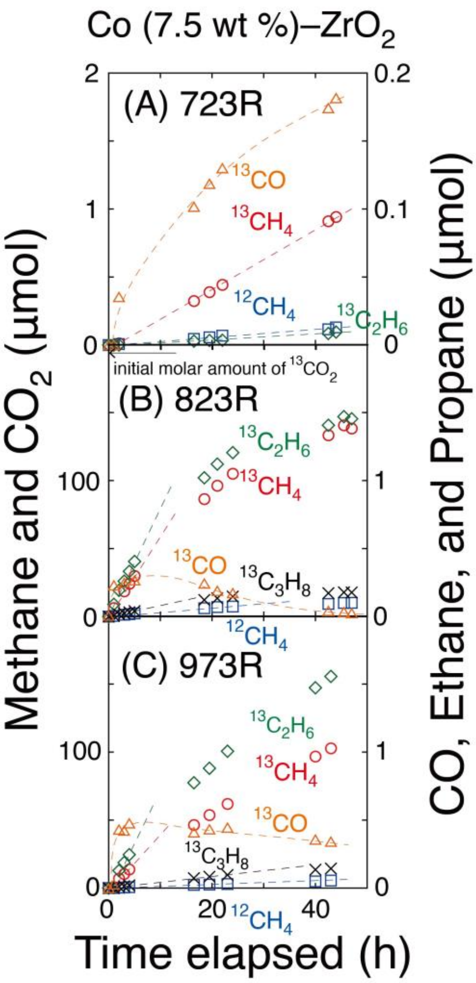

Figure S2. Time–course formation of photocatalytic ^{13}CO, $^{13}CH_4$, $^{12}CH_4$, $^{13}C_2H_6$, and $^{13}C_3H_8$ irradiated under UV–visible light (142 mW cm^{-2}) during exposure to $^{13}CO_2$ (2.3 kPa) and H_2 (21.7 kPa) with Co (7.5 wt %)–ZrO_2 (0.020 g) treated in H_2 at (A) 723 K, (B) 823 K, and (C) 973 K.

WILEY

Table S3. Kinetic Data for Photoconversion of CO_2 and in the Absence of CO_2 Using the H_2-pretreated ZrO_2 and Co–ZrO_2 Photocatalysts Both at 823 K under UV and/or Visible Light Irradiation.

Entry	Catalyst	Reactant-1	Reactant-2	Light irradiated	Formation rate (μmol h^{-1} g$_{cat}^{-1}$)*2 ^{13}CO	$^{13}CH_4$	$^{12}CH_4$	$^{13}C_2H_6$	$^{13}C_3H_8$	Molar ratio of $^{12}CH_4$/($^{13}CH_4$ + $^{12}CH_4$) (%)
a	ZrO_2	$^{13}CO_2$ (2.3 kPa)	H_2 (21.7 kPa)	full light (142 mW cm^{-2})	1.4	< 0.002				–
b			H_2 (2.3 kPa)		1.8					
c	Co (2.5 wt %)–ZrO_2		H_2 (21.7 kPa)		4.1*1	42	3.1	0.63	0.064	7.0
d	Co (5.0 wt %)–ZrO_2				6.1*1	64	7.1	1.2	0.19	9.9
e	Co (7.5 wt %)–ZrO_2				3.6*1	300	23	4.1	0.41	7.1
f		N_2 (2.3 kPa)			< 0.002	0.027*1	1.8*1	< 0.002		98.5
g		$^{13}CO_2$ (2.3 kPa)		> 320 nm	3.5	130	6.0	2.6	3.4	4.4
h				> 520 nm	3.7	67	5.0	0.89	0.099	7.0
i	Co (10 wt %)–ZrO_2			full light	3.2*1	52	4.8	0.65	0.071	8.5

*1 The formation ceased/decreased in 1–4 h of reaction owing to the noncatalytic nature and/or subsequent consecutive reaction.
*2 $^{13}C_2H_4$ and $^{13}C_3H_6$ were not found above the detection limit > 0.002 μmol h^{-1} g$_{cat}^{-1}$.

Table S4. $^{12}C/^{13}C$ Isotopic Ratio on the Photoconversion of CO_2 or CO Using Co (7.5 wt %)–ZrO_2 Photocatalysts under UV–Visible Light Irradiation (142 mW cm^{-2}; Associated Data with Table 1, the main text).

Entry	C oxide	Reductant	*T* (K) under H_2	$^{12}CH_4$/($^{13}CH_4$ + $^{12}CH_4$) (mol-%)
a	$^{13}CO_2$ (2.3 kPa)	H_2 (21.7 kPa)	–	–
b			723	12
c			823	7.1
d			973	4.5
e		H_2 (2.3 kPa)		6.0
f		H_2O (2.2 kPa)	823	17.7
g	^{13}CO (2.3 kPa)	H_2 (2.3 kPa)		6.7
h		H_2 (21.7 kPa)	973	18
i		H_2 (2.3 kPa)		7.9

Table S5. Uptake and Exchange of CO_2 Using ZrO_2-723R and Co–ZrO_2-823R under UV–Visible Light Irradiation (142 mW cm^{-2}).*1

Entry	Catalyst	Physisorption Amount (μmol)	Physisorption Rate constant (h^{-1})	Chemisorption Amount (μmol)	Chemisorption Rate constant (h^{-1})
a	ZrO_2-723R[S20]	9.9	8.0	0.66	0.07
b	Co (7.5 wt %)–ZrO_2-823R	19	4.7	0.050	0.2
		3.9	0.4		
		3.9	0.4		

*1 The amount of photocatalyst was 0.020 g.

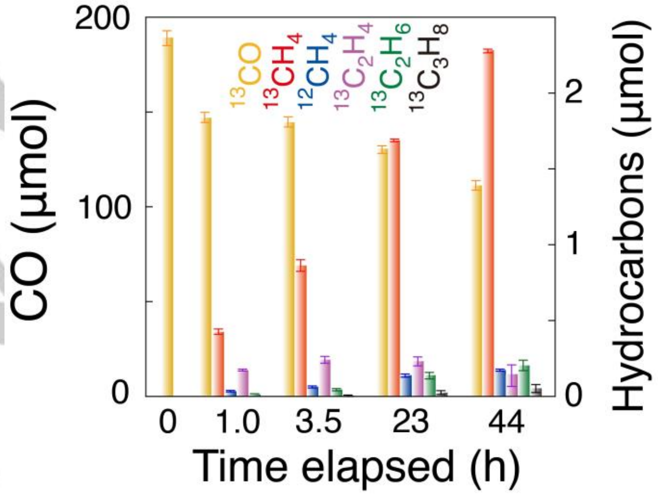

Figure S3. Time–course formation of photocatalytic $^{13}CH_4$, $^{12}CH_4$, $^{13}C_2H_4$, $^{13}C_2H_6$, and $^{13}C_3H_8$ and the decrease of ^{13}CO under UV–visible light exposure (142 mW cm^{-2}) during the presence of ^{13}CO (2.3 kPa) and H_2 (2.3 kPa) with Co (7.5 wt %)–ZrO_2 (0.020 g) catalyst treated in H_2 at 823 K. The error bars for each product were evaluated based on three factors described in *3.1. Kinetic Results*.

Using CO and H_2 as reactants, the consecutive reaction kinetics of C_2H_4 followed by C_2H_6 formation (Figures 2, 3, and S3) was analyzed.

$$2CH_2(\text{surface}) \xrightarrow{k_1} C_2H_4,\ C_2H_4 + 2H(\text{surface}) \xrightarrow{k_2} C_2H_6 \quad \text{(S1)}$$

$$x = [2CH_2],\ y = [C_2H_4],\ z = [C_2H_6] \quad \text{(S2)}$$

$$x_0 = a,\ y_0 = 0,\ z_0 = 0 \quad \text{(S3)}$$

$$\frac{dx}{dt} = -k_1 x,\ \frac{dy}{dt} = k_1 x - k_2 y,\ \frac{dz}{dt} = k_2 y \quad \text{(S4)}$$

$$x = a e^{-k_1 t},\ y = \frac{k_1 a}{k_2 - k_1}\left(e^{-k_1 t} - e^{-k_2 t}\right),\ z = a\left(1 - \frac{k_2}{k_2 - k_1} e^{-k_1 t} + \frac{k_1}{k_2 - k_1} e^{-k_2 t}\right)$$

(S5)

The time–course change of $^{13}C_2H_4$ and $^{13}C_2H_6$ molar amounts fitted well (Figure S4) when the parameters a, k_1, and k_2 were 0.31 μmol, 0.51 h^{-1}, and 0.0077 h^{-1}, respectively. The amount of $^{13}C_2H_6$ formed was 0.26 μmol at 47 h of photoreaction (Figure 2), while the *z* value was 0.090 μmol based on Eq. S5. Thus, 35% of C_2H_6 was generated via C_2H_4 hydrogenation, i.e., Eq. S1, while 65% of C_2H_6 was formed via coupling CH_3 species with CH_2 or CH_3 species over the Co surface.

In summary, selective C_2H_4 formation was enabled when the k_1 value was greater by a factor of 66 times than the k_2 value, while a nonconsecutive route directly forming C_2H_6 via CH_3 coupling was also plausible.

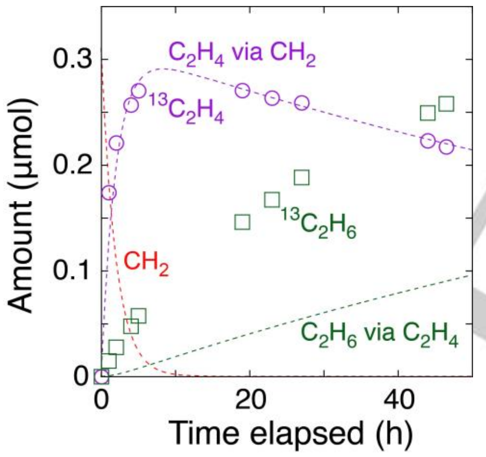

Figure S4. Time–course formation of photocatalytic $^{13}C_2H_4$ and $^{13}C_2H_6$ irradiated with UV–visible light (142 mW cm^{-2}) during exposure to ^{13}CO (2.3 kPa) and H_2 (2.3 kPa) with Co (7.5 wt %)–ZrO_2 (0.020 g) treated in H_2 at 823 K, along with the fit to consecutive reaction via Eq. S5 and calculated amounts of CH_2 species and C_2H_6 via C_2H_4.

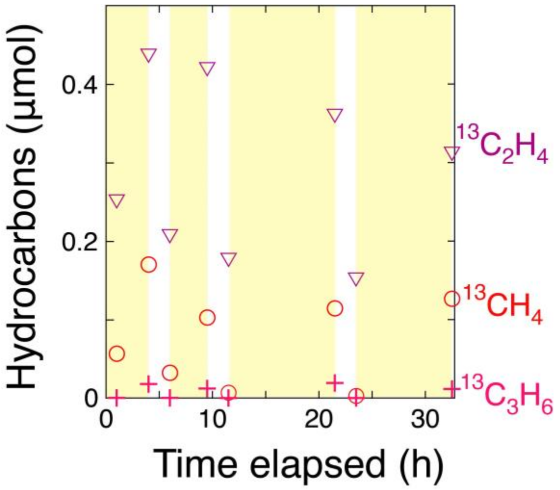

Figure S5. Time–course formation of photocatalytic $^{13}CH_4$, $^{13}C_2H_4$, and $^{13}C_2H_6$ (i) during exposure to ^{13}CO (2.3 kPa) and H_2 (2.3 kPa) for 4–10 h, followed by (ii) 1 h of evaluation and (iii) subsequent exposure to ^{13}CO (2.3 kPa) for 1 h using Co (7.5 wt %)–ZrO_2-973R (0.020 g). Irradiated light intensity 142 mW cm^{-2}. The cycle of steps (i)–(iii) was repeated four times.

A blank test using N_2 (2.3 Pa), H_2 (21.7 kPa), Co (7.5 wt %)–ZrO_2-823R, and UV–visible light irradiation was also performed. Only CH_4 was formed, consistent with the natural abundance ratio of ^{12}C: ^{13}C (98.9: 1.1; Table S3f). However, the time course exhibited noncatalytic behavior, with the formation rate decreasing after 1 h of photoreaction (not shown). The total amount of CH_4 formed in 27 h was 0.06 μmol per 20 mg of photocatalyst, in consistent with the chemisorbed $^{12}CO_2$ amount (0.050 μmol) determined using by $^{13}CO_2$ exchange reaction (Figure 4 and Table S5b).

Notably, the catalytic formation of $^{12}CH_4$ using $^{13}CO_2$ (^{13}CO) includes the contribution of 1.0% of impurity $^{12}CO_2$ (^{12}CO) in the $^{13}CO_2$ (^{13}CO) reagent (see *2. Experimental* section, Tables 1, S3a–e and g–i, S4, and S6).

Control thermal reaction tests were performed using Co (7.5 wt %)–ZrO_2-823R, $^{13}CO_2$ (2.3 kPa), and H_2 (21.7 kPa) at a reaction temperature between 363 and 423 K (Table S6A and Scheme 1D). The formation rate ratio of $^{13}CH_4$, $^{12}CH_4$, $^{13}C_2H_6$, to $^{13}C_3H_8$ was 100: 3.6–11: 0.56–1.1: 0–0.14 compared to those observed in photocatalytic reaction, which were 100: 7.7: 1.4: 0.14 (Table 1c).

Control thermal reaction tests were also performed using Co (7.5 wt %)–ZrO_2-973R, ^{13}CO (2.3 kPa), and H_2 (2.3 kPa) at temperatures between 363 and 423 K (Table S6B and Scheme 1D). At 423 K, the formation rate ratio of $^{13}CH_4$, $^{12}CH_4$, $^{13}C_2H_4$, $^{13}C_2H_6$, $^{13}C_3H_6$, to $^{13}C_3H_8$ was 100: 16: 2.9: 12: 13: 4.5 (Table S6B-c) compared to those observed in photocatalytic reaction, which were 100: 8.6: 243: 11: 34: 1.1 (Table 1i). Notably, olefin formation was especially suppressed in the thermal reaction, suggesting different reaction pathways under UV–visible light irradiation compared to the traditional Fischer–Tropsch mechanism.[S34,S35]

3.2. Characterizations.

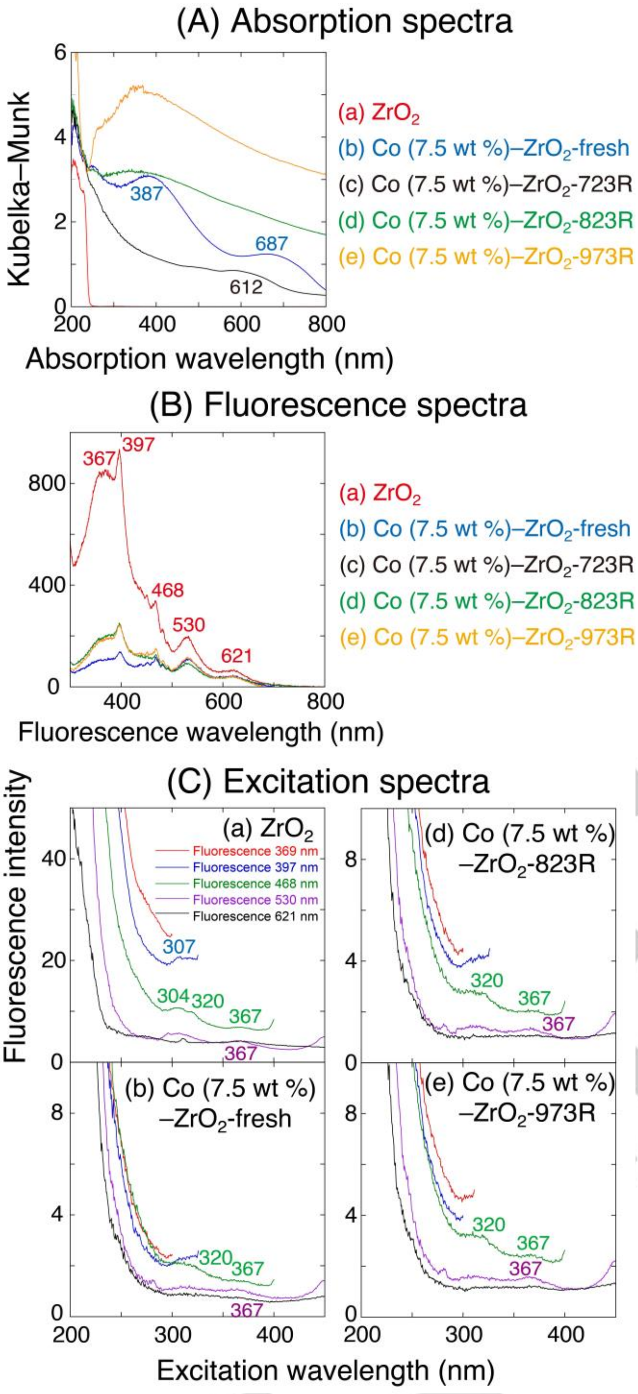

Figure S6. (A) Diffuse-reflectance UV–visible absorption, (B) fluorescence, and (C) excitation spectra of ZrO_2 (a), fresh Co (7.5 wt %)–ZrO_2 (not pretreated with temperature) (b), and Co (7.5 wt %)–ZrO_2 treated in H_2 at 723 K (c), 823 K (d), and 973 K (e). The excitation wavelength is 200 nm in the fluorescence spectra in (B) and the monitored fluorescence wavelength is 369, 397, 468, 530, and 621 nm in the excitation spectra in (C) for photocatalysts a, b, d, and e.

The bandgap value (5.0 eV) obtained based on absorption spectrum (Figure S6A-a) was within the range of reported experimental values.[S36] In the fluorescence spectrum of ZrO_2 excited at 200 nm (Figure S6B-a), the short-wavelength excitation corresponding to near band-edge transitions at 367 and 397 nm was observed, along with excitation from/to the midgap trap states at 468, 530, and 621 nm were observed. The intensity of all the peaks was suppressed to one-tenth to one-fifth for Co (7.5 wt%)–ZrO_2 (Figure S6B-b–e) owing to the charge transfer of light-excited electrons to Co_3O_4, CoO, or metallic Co.

In the excitation spectra for ZrO_2, fluorescence at 397, 468, and 530 nm appeared from the excitation at 304 nm, while fluorescence at 468 and 530 nm appeared from the excitation at 320 nm. Additionally, fluorescence at 468, 530, and 621 nm appeared from the excitation at 367 nm (Figure S6C-a), all attributed to the near band-edge and midgap trap states on/in ZrO_2. These peaks were substantially suppressed owing to the charge transfer effect of Co species (Figure S6C-b, d, and e), consistent with the trend observed in the fluorescence spectra (Figure S6B).

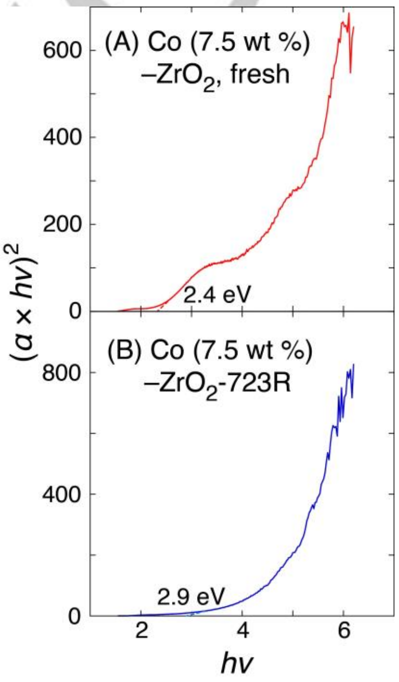

Figure S7. Davis–Mott plots for $n = 1/2$ (allowed direct transition) in Eq. 3 depicting the UV–visible absorption spectra of (A) fresh Co (7.5 wt %)–ZrO_2 (not pretreated with temperature) and (B) Co (7.5 wt %)–ZrO_2-723R.

The valence band maximum (E_{VBM} vs. normal hydrogen electrode (NHE)) was calculated based on the work function of the XPS apparatus (4.23 eV) and the XPS binding energy (Figure S8C).

4.23 + 2.54 − 4.44 = 2.33 V for Co (7.5 wt %)–ZrO_2-723R

4.23 + 2.62 − 4.44 = 2.41 V for Co (7.5 wt %)–ZrO_2-823R

The E_g value was 2.89 eV based on the Davis–Mott equation[S37] ($n = 1/2$) for CoO (Figure S7B). Thus,

E_{CBM} vs. NHE

= E_{VBM} vs. NHE – E_g

= 2.33 − 2.89 = −0.56 V for Co (7.5 wt %)–ZrO_2-723R

= 2.41 − 2.89 = −0.48 V for Co (7.5 wt %)–ZrO_2-823R

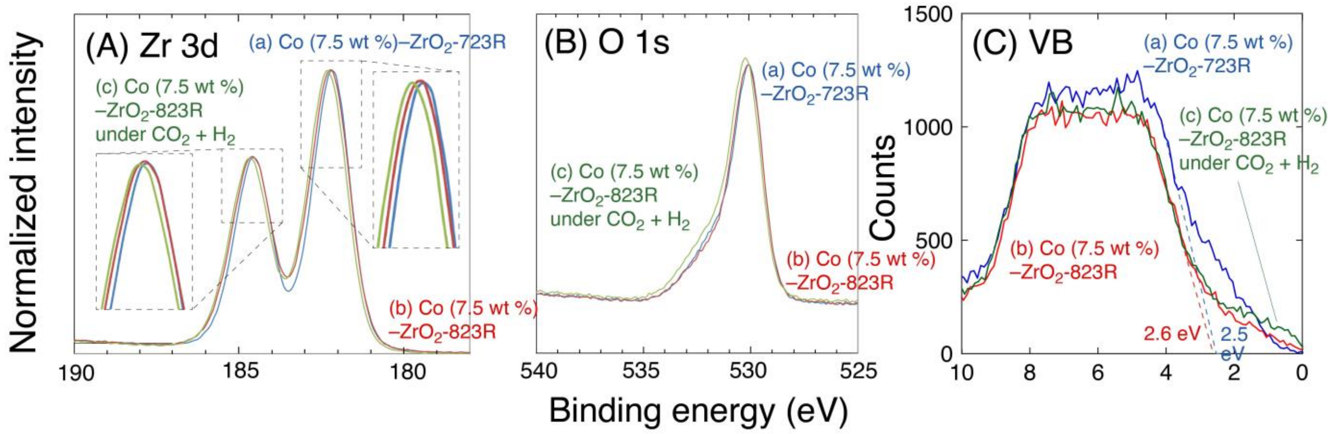

Figure S8. XPS spectra in (A) Zr 3d, (B) O 2s, and (C) valence band regions measured for (a) Co (7.5 wt %)–ZrO_2-723R, (b) Co (7.5 wt %)–ZrO_2-823R, and (c) Co (7.5 wt %)–ZrO_2-823R under 2.3 kPa of CO_2 and 21.7 kPa of H_2. Expanded view of peak tops is also drawn in (A).

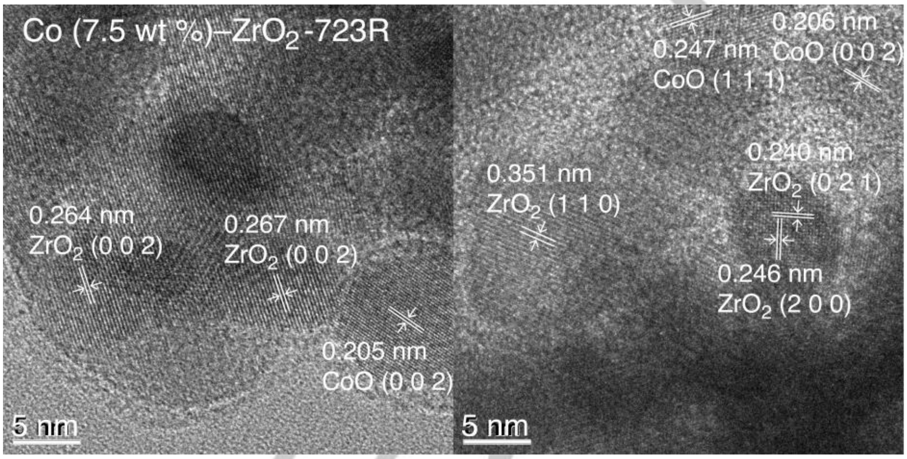

Figure S9. HR-TEM images of Co (7.5 wt%)–ZrO_2-723R photocatalyst. Lattice intervals for monoclinic ZrO_2 and CoO were also drawn.

Table S6. Control Kinetic Data for Thermal Conversion at Reaction Temperature 363–423 K (A) Using $^{13}CO_2$ (2.3 kPa), H_2 (21.7 kPa), and the H_2-pretreated Co (7.5 wt %)–ZrO_2 Catalyst (0.020 g) at 823 K and (B) Using ^{13}CO (2.3 kPa), H_2 (2.3 kPa), and the H_2-pretreated Co (7.5 wt %)–ZrO_2 Catalyst (0.020 g) at 973 K in the Absence of UV–Visible Light Irradiation.

(A) $^{13}CO_2$ (2.3 kPa), H_2 (21.7 kPa), and Co (7.5 wt %)–ZrO_2-823R										
Entry	$T_{reaction}$ (K)	Formation rate (μmol h^{-1} g_{cat}^{-1})								Molar ratio of $^{12}CH_4$/ ($^{13}CH_4$ + $^{12}CH_4$) (%)
		^{13}CO	$^{13}CH_4$	$^{12}CH_4$	$^{13}C_2H_4$	$^{13}C_2H_6$	$^{13}C_3H_6$	$^{13}C_3H_8$	Σ_C	
a	363	0.006*1	1.6	0.18		0.009		<0.002	1.8	10
b	393	0.085*1	13	0.48	<0.002	0.081	<0.002	0.018	14	3.5
c	423	8.5*1	79	3.4		0.87		0.040	91	4.2
(B) ^{13}CO (2.3 kPa), H_2 (2.3 kPa), and Co (7.5 wt %)–ZrO_2-973R										
a	363	<0.002								-
b	393	-	0.026	0.0071	<0.002				0.035	21
c	423	-	0.63	0.10	0.019	0.075	0.081	0.029	0.94	14

*1 The formation ceased/decreased in 1–3 h of reaction owing to the noncatalytic nature and/or subsequent consecutive reaction.

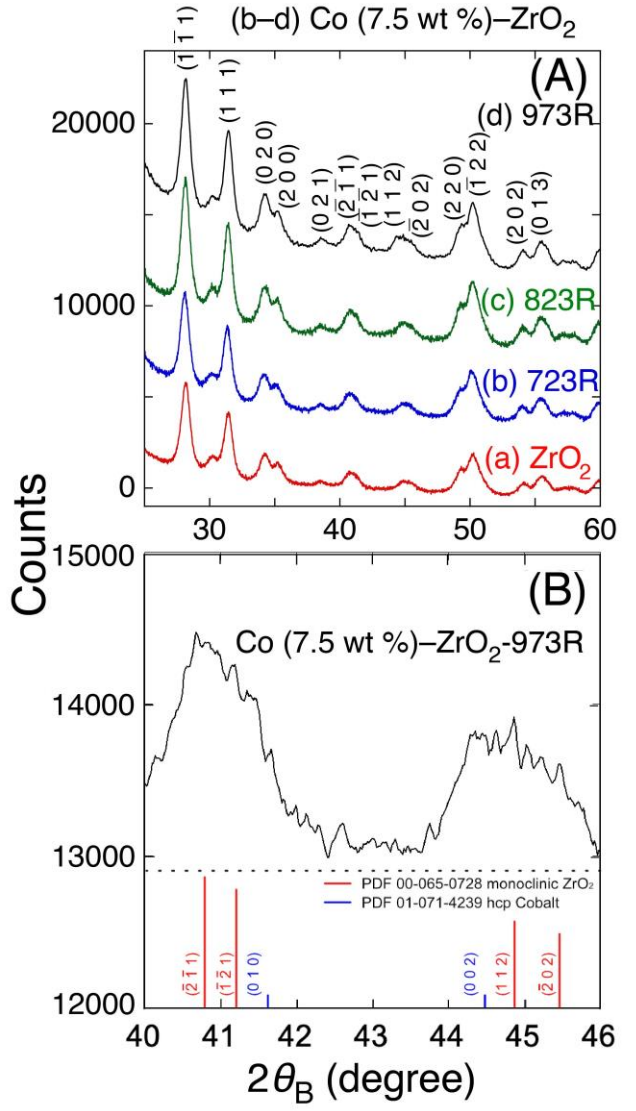

Figure S10. XRD patterns of (A) (a) ZrO_2, Co (7.5 wt %)–ZrO_2 treated in H_2 at (b) 723 K, (c) 823 K, and (d) 973 K with peak assignments and (B) expanded pattern with peak assignments for the data in panel A-d.

The Co K-edge XANES spectra were measured (Figure S11A) to identify the Co species. The XANES pattern for fresh Co (7.5 wt %)–ZrO_2, Co (7.5 wt %)–ZrO_2-723R, and Co (7.5 wt %)–ZrO_2-973R resembled those of standard samples of Co_3O_4, CoO, and Co metal, respectively.

For Co (7.5 wt %)–ZrO_2-823R, the XANES pattern was fit using the convolution of the XANES pattern by varying the mixing ratio between the normalized XANES spectra of metallic Co and CoO. The best fit was achieved with a ratio of 0.85: 0.15 (Figure S11B). Interestingly, the surface Co^0 site (~40% of total Co sites) of metallic Co nanoparticles slowly oxidized to Co^{II} in the presence of CO_2 and H_2 gas for 1 day without UV–visible light irradiation (Figure S11B).

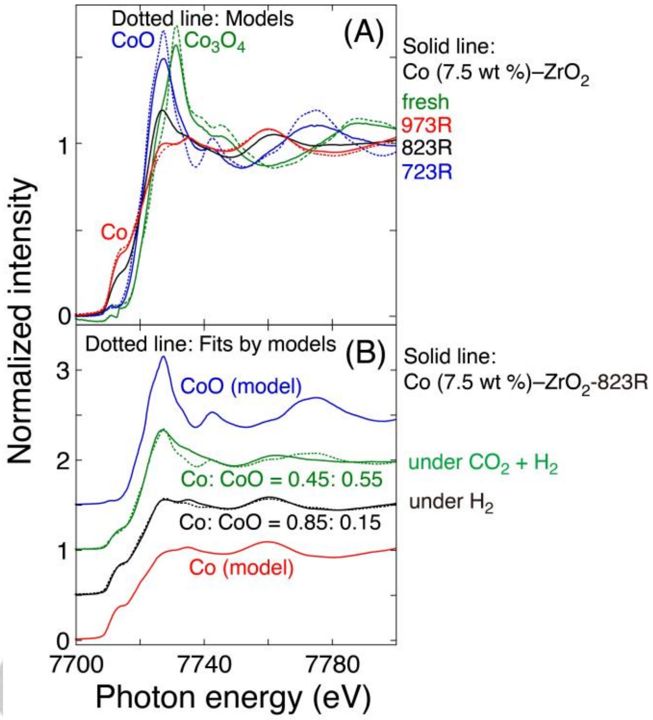

Figure S11. Normalized Co K-edge XANES spectra for (A) the standard Co metal, CoO, and Co_3O_4 samples (dotted lines) compared to the fresh Co (7.5 wt %)–ZrO_2, Co (7.5 wt %)–ZrO_2-723R, Co (7.5 wt %)–ZrO_2-823R, and Co (7.5 wt %)–ZrO_2-973R photocatalysts (solid lines), and (B) Co metal, CoO, and Co (7.5 wt %)–ZrO_2-823R under H_2 (21.7 kPa) only and under a mixture of CO_2 (2.3 kPa) and H_2 (21.7 kPa) (solid lines), along with the convolution of normalized XANES spectra for Co and CoO with the mixing ratios of 0.85: 0.15 and 0.45: 0.55 (dotted lines).

The major CH_4 formation rate reached 196 μmol h^{-1} g_{cat}^{-1} during the initial 10 min of UV–visible light irradiation over the Co (7.5 wt %)–ZrO_2-973R photocatalyst (20 mg), resulting in the formation of 0.65 μmol of CH_4. Calculating based on the standard reaction enthalpy of CO_2 hydrogenation to CH_4 (−164.94 kJ mol^{-1}), the heat of the reaction was determined to be 0.11 J. Based on the molar heat capacity of Co and ZrO_2 (24.81 J K^{-1} mol^{-1} [S38] and 56.123 J K^{-1} mol^{-1},[S39] respectively), the resulting temperature rise was calculated at 11.9 K. However, this increase was notably smaller than the observed initial temperature increase of 66 K in 10 min (Figure 6). Thus, the temperature elevation was attributed to the transformation of light energy into heat at the Co^0 surface, which quickly reached the equilibrium (Figure 6), dissipating heat into the reactor/XAFS cell.

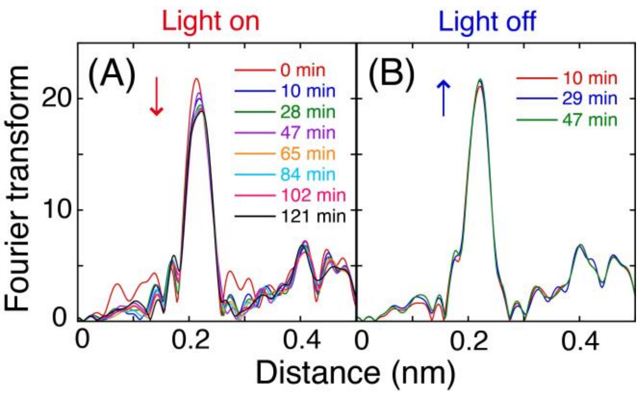

Figure S12. Time–course evolution of the Fourier transform of the angular wave number k^3-weighted EXAFS χ-function for the Co (7.5 wt %)–ZrO_2-973R photocatalyst under CO_2 (2.3 kPa) and H_2 (21.7 kPa) (A) during UV–visible light irradiation (142 mW cm^{-2}) and (B) under dark.

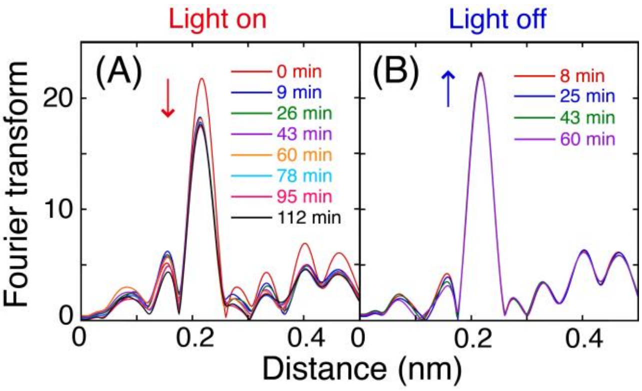

Figure S14. Time–course evolution of the Fourier transform of the angular wave number k^3-weighted EXAFS χ-function for the Co (7.5 wt %)–ZrO_2-973R photocatalyst under CO (2.3 kPa) and H_2 (2.3 kPa) (A) during UV–visible light irradiation (142 mW cm^{-2}) and (B) under dark.

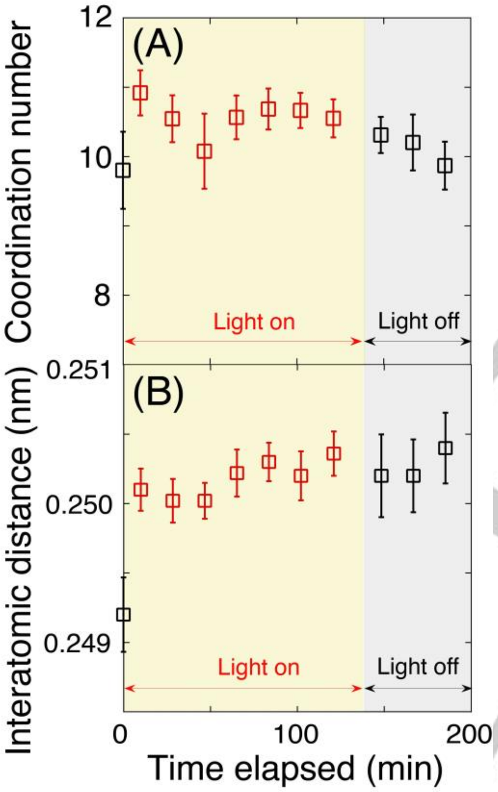

Figure S13. Time course analysis of (A) coordination number (*N*) and (B) interatomic distance (*R*) derived from Co K-edge EXAFS analysis for Co (7.5 wt %)–ZrO_2-973R using CO_2 (2.3 kPa), H_2 (21.7 kPa), and UV–visible light irradiation (142 mW cm^{-2}) using curve-fit analyses and under dark. The error bars were drawn based on data reproducibility in three runs and the fit errors.

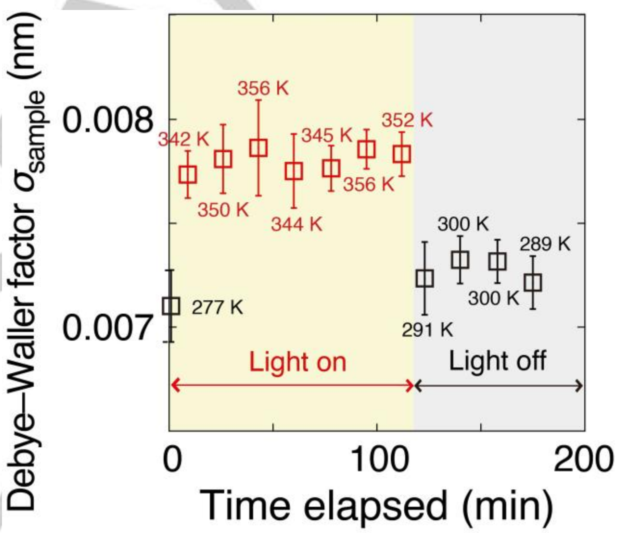

Figure S15. Time course analysis of Debye–Waller factor derived from Co K-edge EXAFS analysis and the corresponding temperature evaluation based on the correlated Debye model of Co nanoparticles in Co (7.5 wt %)–ZrO_2-973R using CO (2.3 kPa), H_2 (2.3 kPa), and UV–visible light irradiation (142 mW cm^{-2}) and under dark. The error bars were drawn based on data reproducibility in three runs and the fit errors.

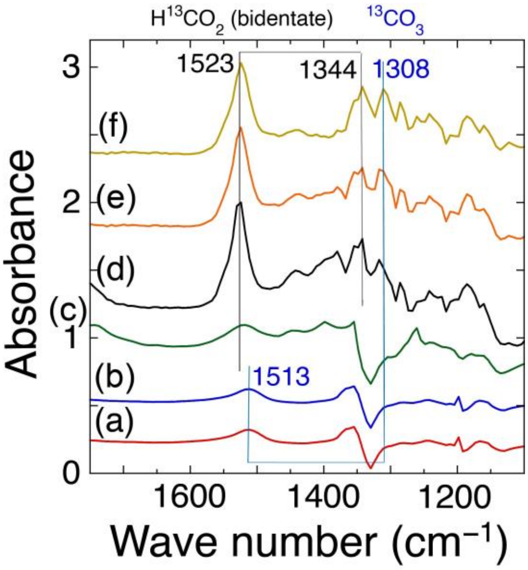

Figure S16. FTIR monitoring of the O–C–O stretching vibration region and the peak assignments using a Co (7.5 wt %)–ZrO_2-973R photocatalyst (71 mg). The conditions were as follows: (a) under ^{13}CO (2.3 kPa) and H_2 (2.3 kPa) in the dark for 2 h, (b–d) under ^{13}CO (2.3 kPa), H_2 (2.3 kPa), and UV–visible light irradiation at 0 h (b), 12 h (c), and 34 h (d), and (e, f) under vacuum and UV–visible light irradiation at 0 h (e) and 2 h (f).

3.3. DFT Calculations. The photocatalytic CO_2 reduction reaction using Co (7.5 wt %)–ZrO_2 proceeded steadily (Figure S2), mostly owing to the effective separation of $V_O^{\cdot\cdot}$ sites for CO_2 adsorption/activation and Co^0 sites for H_2 activation/hydrogenation (Schemes 1A and 2A). Notably, the activation energy from CH_2 to CH_3 species decreased from 0.55 eV in the absence of neighboring H atoms to 0.30 eV (Scheme 2A-d) in the presence of neighboring H atoms over the Co^0 surface.

Furthermore, the photocatalytic CO reduction reaction proceeded consecutively (Figures 2, S3, and S4). The $V_O^{\cdot\cdot}$ site became occupied by hydroxy species and the concentration of H species over Co^0 sites gradually increased, making CO adsorption on Co^0 became unfavorable and leading to hydrogenation of C_2H_4 species (Scheme 2B-b′) ultimately resulting in paraffin formation.

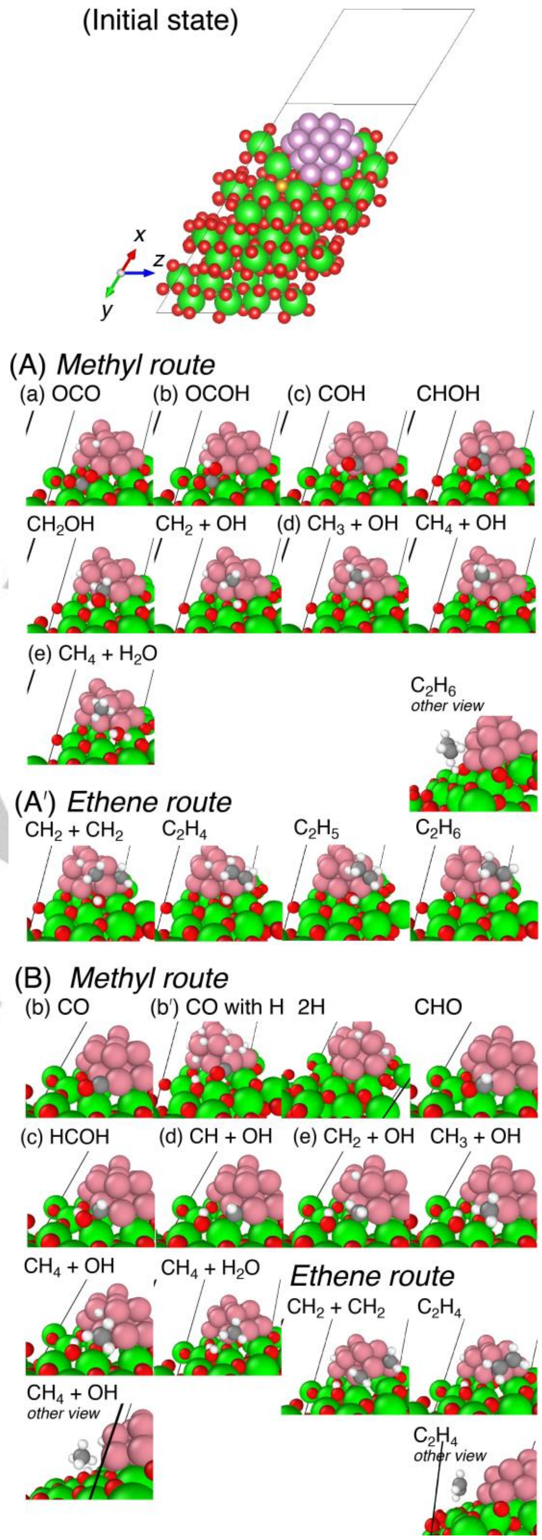

Scheme S2. Three-Dimensional Illustration of Surface Species (Scheme 2A, A', and B) over a Monoclinic ZrO_2 (1 1 1) Surface, Combined with a Co_{19} Nanocluster Exposing (0 1 0) Surface. The Illustration Created Using the VESTA Version 3.5.7[S40] (Initial State), and (A, A') under CO_2 and H_2 and (B) under CO and H_2, Using the OVITO Version 3.7.8.[S41] Green, Zr Atom; Red, O Atom; Gold (the Panel of Initial State), O Atom to Be Removed Bound to Two Zr Atoms;[S33] Gray, C Atom; White, H Atom; Purple, Co Atom.

WILEY

3.4. Photocatalytic Conversion of CO_2 Using H_2O. The results of the CO_2 photoreduction test in liquid H_2O are summarized in Table S8. Under UV–visible light irradiation (90.2 mW cm^{-2} per flask, Chart S2), CO_2 photoconversion was very limited, e.g. 7.2 μmol h^{-1} g_{cat}^{-1} of CO and CH_4 formed in total (Table S8c) or 0.095 μmol h^{-1} g_{cat}^{-1} of C_2H_6 and C_3H_8 formed in total (Table S8d). In contrast, H_2O photosplitting proceeded well at the formation rates of 23–690 μmol h^{-1} g_{cat}^{-1} using ZrO_2 and Co (7.5 wt %)–ZrO_2-973R photocatalysts (Table S8a–e).

With increase in light intensity from 90.2 to 222 mW cm^{-2} per flask (Chart S2), the formation rates of C_1, C_2, and C_3 products increased to 22–50, 1.5–1.6, and 0.52–1.0 μmol h^{-1} g_{cat}^{-1}, respectively, using H_2O (70 mL) and either Co (7.5 wt %)–ZrO_2-823R or -973R photocatalyst (Table S8f and g and Figure 8D).

3.5. Photocatalytic Conversion of $^{13}CO_2$ Using D_2O. The photoreduction of $^{13}CO_2$ (2.3 kPa) using D_2O (2.2 kPa), H_2 (21.7 kPa), and Co (7.5 wt %)–ZrO_2-823R formed $^{13}CH_4$, $^{13}CH_3D$, $^{13}CH_2D_2$, $^{13}CHD_3$, to $^{13}CD_4$ with a ratio of 100: 41: 6.2: 0.21: ~0 (9.2 mol%-D in these products), which agrees with the theoretical H/D distribution: 100: 40: 6.1: 0.41: 0.010 in reactants (9.1 mol%-D in these products; Figure S19 and Table S7b). This suggests that D_2O and H_2 reached equilibrium more rapidly over Co^0 more effectively than the progressive hydrogenation to $C_{1–3}$ paraffin (Scheme 1A-a–d).

The methane formation rate was the 9.6% of that observed using H_2 (Table 1c, e) owing to the competitive adsorption of OH on the Co^0 surface and/or H_2O adsorption at the $V_O^{\cdot\cdot}$ site of ZrO_2. However, during photocatalytic CO_2 reduction tests under H_2O (2.3 kPa; Figure S20) via UV–visible spectroscopy and tests under H_2O (2.3 kPa) and H_2 (21.7 kPa; Figure S21) through Co K-edge EXAFS, the Co^{II} species could not be detected. This demonstrates that the metallic Co surface effectively activates H_2O for $C_{2,3}$ paraffin photosynthesis using either H_2, H_2O (g), or H_2O (l).

Based on the criteria for evaluating the fragment peak of *m*/*z* = 17 ($^{13}CH_4^+$), 16 ($^{12}CH_4^+$), 30 ($^{13}C_2H_4^+$), 30 ($^{13}C_2H_4^+$), 44 ($^{13}C_3H_5^+$), and 31 ($^{13}C_2H_5^+$) for the formation of $^{13}CH_4$, $^{12}CH_4$, $^{13}C_2H_4$, $^{13}C_2H_6$, $^{13}C_3H_6$, to $^{13}C_3H_8$ (see the *2. Methods* section), only the $^{13}CH_3^+$ fraction was considered for *m*/*z* = 16. The contributions of the $^{12}CH_3D^+$ fraction for *m*/*z* = 17 (methane), $^{13}C_2H_2D^+$ for *m*/*z* = 30 (ethene), $^{13}C_2H_2D^+$ and $^{13}C^{12}CH_5^+$ for *m*/*z* = 30 (ethane), $^{13}C_3H_3D^+$ and $^{13}C_2{}^{12}CH_6^+$ for *m*/*z* = 44 (propene), and $^{13}C_2H_3D^+$ and $^{13}C^{12}CH_6^+$ for *m*/*z* = 31 (propane) were all neglected owing to the low D and ^{12}C ratio in the reactant (D: 9.1 mol%) and the inability of our MS spectrometer (Model JMS-Q1050GC, JEOL) to distinguish the mass number after the decimal point. Thus, the tentatively obtained formation molar ratio of ($^{13}CH_4$ + $^{13}CH_3D$ + $^{13}CH_2D_2$ + $^{13}CHD_3$ + $^{13}CD_4$), $^{12}CH_4$, $^{13}C_2H_4$, $^{13}C_2H_6$, $^{13}C_3H_6$, to $^{13}C_3H_8$ was 100: 10: ~0: 1.2: ~0: 1.7, similar to the ratio 100: 7.7: ~0: 1.4: ~0: 0.14 using reactant $^{13}CO_2$ (2.3 kPa) and H_2 (21.7 kPa) (Tables 1c and S7a and b).

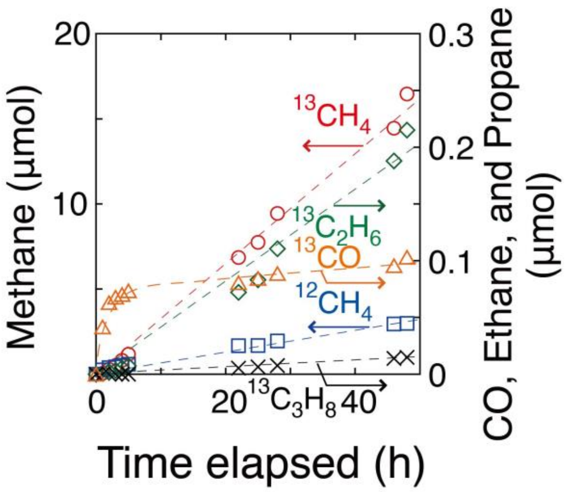

Figure S17. Time–course formation of photocatalytic ^{13}CO, $^{13}CH_4$, $^{12}CH_4$, $^{13}C_2H_6$, and $^{13}C_3H_8$ during exposure to $^{13}CO_2$ (2.3 kPa) and H_2O (2.2 kPa) using Co (7.5 wt %)–ZrO_2-823R, with rapid evacuation of gas phase H_2. Irradiated light intensity 142 mW cm^{-2}.

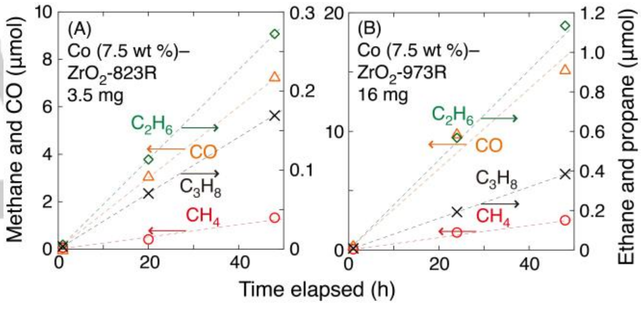

Figure S18. Time–course formation of photocatalytic CO, CH_4, C_2H_6, and C_3H_8 during exposure to CO_2 (95 kPa) and H_2O (70 mL) under UV–visible light irradiation (222 mW per flask; Chart S2) using Co (7.5 wt %)–ZrO_2-823R (3.5 mg; A) and -973R (16 mg; B).

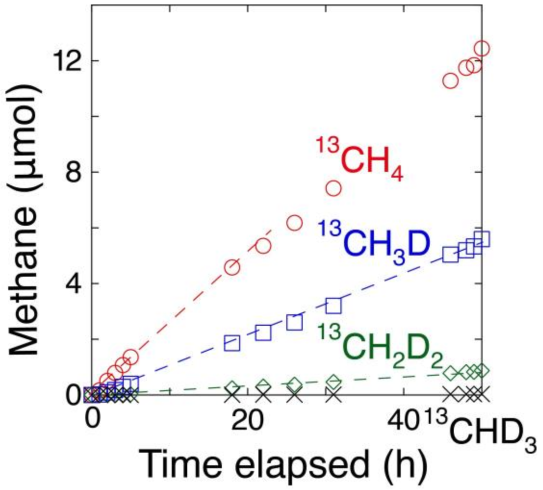

Figure S19. Time–course formation of photocatalytic $^{13}CH_4$, $^{13}CH_3D$, $^{13}CH_2D_2$, and $^{13}CHD_3$ during exposure to $^{13}CO_2$ (2.3 kPa), D_2O (2.2 kPa), and H_2 (21.7 kPa) under UV–visible light irradiation (142 mW cm^{-2}). using Co (7.5 wt %)–ZrO_2-823R. Catalyst amount: 0.020 g.

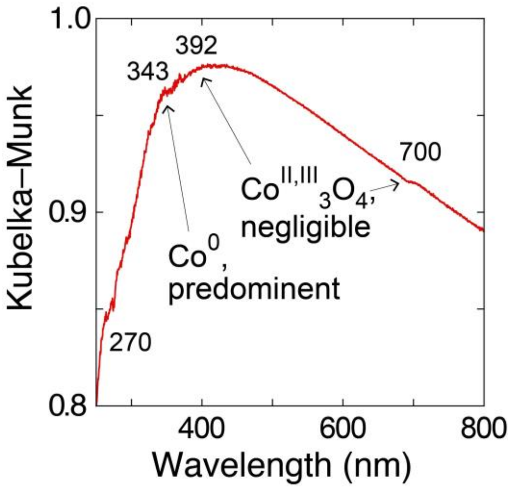

Figure S20. Diffuse-reflectance UV–visible spectrum of Co (7.5 wt %)–ZrO_2-823R photocatalyst (0.088 g) under UV–visible light irradiation (142 mW cm^{-2}) for 72 h, in the presence of CO (2.3 kPa) and H_2O (2.3 kPa). Peak assignment was based on corresponding spectra in Figures S6A-b and e. The broad absorption in the visible light region is attributed to metallic Co^0 species.

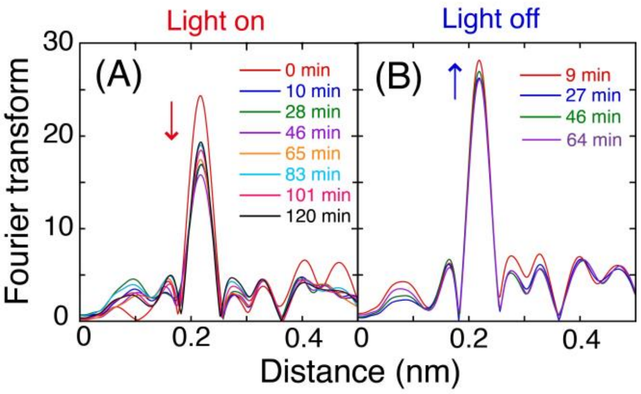

Figure S21. Time–course change of the Fourier transform of the angular wave number k^3-weighted EXAFS χ-function for the Co (7.5 wt %)–ZrO_2-973R photocatalyst under CO_2 (2.3 kPa), H_2O (2.3 kPa), and H_2 (21.7 kPa) (A) during irradiation under UV–visible light (142 mW cm^{-2}) and (B) under dark.

Table S7. Kinetic Data for Photoconversion of CO_2 in the Presence/Absence of D_2O Using H_2 and the H_2-pretreated Co (7.5 wt %)–ZrO_2 Photocatalyst (0.020 g) at 823 K under UV–Visible Light Irradiation (142 mW cm^{-2}).

Entry	Reactants	Formation rate (μmol h^{-1} g$_{cat}{}^{-1}$)								D molar ratio in $^{13}CH_4$ (%)
		$^{13}CH_4$	$^{13}CH_3D$	$^{13}CH_2D_2$	$^{13}CHD_3$	$^{13}CD_4$	$^{13}C_2H_6$	$^{13}C_3H_8$	Σ_C	
a	$^{13}CO_2$ (2.3 kPa) + H_2 (21.7 kPa)	300	–				4.1	0.41	303	–
b	$^{13}CO_2$ (2.3 kPa) + D_2O (2.2 kPa) + H_2 (21.7 kPa)	13	5.4	0.82	0.028	< 0.002	0.23	0.32	20	9.2

Table S8. Kinetic Data for Photoconversion of CO_2 (95 kPa) Using H_2O (70 mL) and ZrO_2 or the H_2-pretreated Co–ZrO_2 Photocatalyst at 823 K or 973 K under UV–Visible Light Irradiation.

Entry	Catalyst		H_2O	Light intensity	Formation rate (μmol h^{-1} g$_{cat}{}^{-1}$)							
	Catalyst	Amount (mg)			CO	CH_4	HCO_2^-	CH_3OH	$^{12}C_2H_6$	$^{12}C_3H_8$	H_2	O_2
a	ZrO_2	5.0	70 mL	90.2 mW per flask	0.68	< 0.008	< 12	< 7.6	–	–	1400	690
b		10			0.37	< 0.008	< 6.2	< 3,8	–	–	630	320
c	Co (7.5 wt %)–ZrO_2-973R	2.2			3.6	3.6	< 28	< 17	–	–	2700	520
d		9.0			0.90	0.25	< 6.9	< 4.2	0.071	0.024	580	110
e		37			0.74	0.064	< 1.7	< 1.0	–	–	150	23
					^{12}CO	$^{12}CH_4$	HCO_2^-	CH_3OH	$^{12}C_2H_6$	$^{12}C_3H_8$	$^1H_2$	$^{16}O_2$
f	Co (7.5 wt %)–ZrO_2-823R	3.5	70 mL	222 mW per flask	41	8.8	< 18	< 11	1.6	1.0	1300	590
g	Co (7.5 wt %)–ZrO_2-973R	16			19	3.4	< 3.9	< 2.4	1.5	0.52	500	66

Corresponding Author

* yizumi@faculty.chiba-u.jp

ORCID

T. Loumissi: 0000-0002-4840-8625
R. Ishii: 0000-0001-7224-7107
K. Hara: 0000-0002-5014-9079
T. Oyumi: 0000-0002-8339-5161
I. Abe: 0009-0008-9732-6846
C. Li: 0009-0008-1494-2708
H. Zhang: 0000-0002-6773-8769
R. Hirayama: 0000-0002-9706-3010
K. Niki: 0000-0002-0795-1007
T. Itoi: 0000-0002-2864-6650
Y. Izumi: 0000-0001-8366-1864

Acknowledgements

The authors thank Daiki Mitsumoto, Chiba University for NMR measurements.

[S1] N. Li, B. Wang, Y. Si, F. Xue, J. Zhou, Y. Lu, M. Liu, *ACS Catal.* **2019**, *9*(6), 5590–5602.
[S2] H. Ou, G. Li, W. Ren, B. Pan, G. Luo, Z. Hu, D. Wang, *J. Am. Chem. Soc.* **2022**, *144*(48), 22075–22082.
[S3] H. Park, H.-H. Ou, A. J. Colussi, M. R. Hoffmann, *J. Phys. Chem. A* **2015**, 119(19), 4658–4666.
[S4] W. Kim, T. Seok, W. Choi, *Energy Environ. Sci.* **2012**, *5*(3), 6066–6070.
[S5] S. Sorcar, J. Thompson, Y. Hwang, Y. H. Park, T. Majima, C. A. Grimes, J. R. Durrant, *Energy Environ. Sci.* **2018**, *11*(11), 3183–3193.
[S6] W. Tu, Y. Zhou, Q. Liu, S. Yan, S. Bao, X. Wang, M. Xiao, Z. Zou, *Adv. Funct. Mater.* **2013**, *23*(14), 1743–1749.
[S7] Y. Wang, J. Zhao, Y. Liu, G. Liu, S. Ding, Y. Li, J. Xia, H. Li, *J. Colloid Interf. Sci.* **2022**, *616*, 649–658.
[S8] X. Zhang, F. Han, B. Shi, S. Farsinezhad, G. P. Dechaine, K. Shankar, *Angew. Chem. Int. Ed.* **2012**, *51*(51), 12732–12735.
[S9] Q. Chen, X. Chen, M. Fang, J. Chen, Y. Li, Z. Xie, Q. Kuang, L. Zheng, *J. Mater. Chem.* A **2019**, 7(3), 1334–1340.
[S10] B. Ni, G. Zhang, H. Wang, Y. Min, K. Jiang, H. Li, *Angew. Chem. Int. Ed.* **2023**, *62*(6), e202215574.
[S11] L. Yu, G. Li, X. Zhang, X. Ba, G. Shi, Y. Li, P. K. Wong, J. C. Yu, Y. Yu, *ACS Catal.* **2016**, *6*(10), 6444–6454.
[S12] K. Adachi, K. Ohta, T. Mizuno, *Solar Ener.* **1994**, *53*(2), 187–190.
[S13] M. M. Gui, W. M. P. Wong, S.-P. Chai, A. R. Mohamed, *Chem. Eng. J.* **2015**, *278*, 272–278.
[S14] S. Chakraborty, R. Das, M. Riyaz, K. Das, A. K. Singh, D. Bagchi, C. P. Vinod, S. C. Peter, *Angew. Chem. Int. Ed.* **2023**, *62*(9), e202216613.
[S15] K. Yan, D. Wu, T. Wang, C. Chen, S. Liu, Y. Hu, C. Gao, H. Chen, B. Li, *ACS Catal.* **2023**, *13*(4), 2302–2312.
[S16] Y. Wu, Q. Chen, J. Zhu, K. Zheng, M. Wu, M. Fan, W. Yan, J. Hu, J. Zhu, Y. Pan, X. Jiao, Y. Sun, Y. Xie, *Angew. Chem. Int. Ed.* **2023**, e202301075.
[S17] R. Das, R. Paul, A. Parui, A. Shrotri, C. Atzori, K. A. Lomachenko, A. K. Singh, J. Mondal, S. C. Peter, *J. Am. Chem. Soc.* **2023**, *145*(1), 422–435.
[S18] F. Guo, R.-X. Li, S. Yang, X.-Y. Zhang, H. Yu, J. J. Yuban, W.-Y. Sun, *Angew. Chem. Int. Ed.* **2023**, *62*, e202216232.
[S19] T. Wang, L. Chen, C. Chen, M. Huang, Y. Huang, S. Liu, B. Li, *ACS Nano* **2022**, *16*(2), 2306–2318.
[S20] H. Zhang, T. Itoi, T. Konishi, Y. Izumi, *Angew. Chem. Int. Ed.* **2021**, *60*, 9045–9054.
[S21] S. Wang, B. Jiang, J. Henzie, F. Xu, C. Liu, X. Meng, S. Zou, H. Song, Y. Pan, H. Li, J. Yu, H. Chen, J. Ye, *Nat. Commun.* **2023**, *14*, 2534.
[S22] H. Zhang, T. Itoi, T. Konishi, Y. Izumi, *J. Am. Chem. Soc.* **2019**, *141*, 6292–6301.
[S23] M. Vaarkamp, H. Linders, D. Koningsberger, XDAP Version 3.2.9.; XAFS Services International: Woudenberg, The Netherlands, 2022.
[S24] https://www.webelements.com/cobalt/crystal_structure.html (checked on June 21, 2024).
[S25] W. Jauch, M. Reehuis, H. J. Bleif, F. Kubanek, *Phys. Rev. B* **2001**, *64*, 052102.
[S26] L. Ankudinov, B. Ravel, J. J. Rehr, S. D. Condradson, *Phys. Rev. B Condens. Matter Mater. Phys.* **1998**, *58*, 7565–7576.
[S27] G. Kresse, J. Furthmüller, *Phys. Rev. B* **1996**, *54*, 11169−11186.
[S28] S. Grimme, S. Ehrlich, L. Goerigk, *J. Comput. Chem.* **2011**, *32*, 1456−1465.
[S29] B. Hammer, L. B. Hansen, J. K. Nørskov, *Phys. Rev. B* **1999**, *59*, 7413− 7421.
[S30] J. Hubbard, *Proc. Royal Soc. A* **1963**, *276*(1365) 238–257.
[S31] V. I. Anisimov, J. Zaanen, O. K. Andersen, *Phys. Rev. B* **1991**, *44*(3), 943–954.
[S32] G. Henkelman, H. Jonsson, *J. Chem. Phys.* **2000**, *113*, 9978−9985.
[S33] K. Hara, M. Nozaki, R. Hirayama, R. Ishii, K. Niki, Y. Izumi, *J. Phys. Chem. C* **2023**, *127*(4), 1776–1788.
[S34] Z. Yao, C. Guo, Y. Mao, P. Hu, *ACS Catal.* **2019**, *9*(7), 5957–5973.
[S35] D. Santos-Carballal, A. Cadi-Essadek, N. H. de Leeuw, *J. Phys. Chem. C* **2021**, *125*(22), 11891–11903.
[S36] H. Jiang, R. I. Gomez-Abal, P. Rinke, M. Scheffler, *Phys. Rev. B* **2010**, *81*, 085119.
[S37] N. Ahmed, Y. Shibata, T. Taniguchi, Y. Izumi, *J. Catal.* **2011**, *279*, 123–135.
[S38] *CRC Handbook of Chemistry and Physics*, 96th ed. (W. M. Haynes, Ed.), CRC Press: Boca Raton, 2015; p 4-124, 12-122.
[S39] M. W. Chase Jr., *NIST-JANAF Themochemical Tables*, 4th Ed., J. Phys. Chem. Ref. Data, Monograph 9 (1998), pp. 1–1951.
[S40] K. Momma, F. Izumi, *J. Appl. Cryst.* **2011**, *44*, 1272–1276.
[S41] A. Stukowski, *Modelling Simul. Mater. Sci. Eng.* **2010**, *18*(1), 015012.

WILEY-VCH
